# Dynamic switching of transcriptional regulators between two distinct low-mobility chromatin states

**DOI:** 10.1126/sciadv.ade1122

**Published:** 2023-06-14

**Authors:** Kaustubh Wagh, Diana A. Stavreva, Rikke A. M. Jensen, Ville Paakinaho, Gregory Fettweis, R. Louis Schiltz, Daniel Wüstner, Susanne Mandrup, Diego M. Presman, Arpita Upadhyaya, Gordon L. Hager

**Affiliations:** ^1^ Laboratory of Receptor Biology and Gene Expression, National Cancer Institute, National Institutes of Health, Bethesda, MD 20892, USA.; ^2^ Department of Physics, University of Maryland, College Park, MD 20742, USA.; ^3^ Department of Biochemistry and Molecular Biology, University of Southern Denmark, Odense, Denmark.; ^4^ Institute of Biomedicine, University of Eastern Finland, Kuopio, P.O. Box 1627, 70211 Kuopio, Finland.; ^5^ Instituto de Fisiología, Biología Molecular y Neurociencias (IFIBYNE), CONICET-Universidad de Buenos Aires, Facultad de Ciencias Exactas y Naturales, Buenos Aires C1428EGA, Argentina.; ^6^ Institute for Physical Science and Technology, University of Maryland, College Park, MD 20742, USA.

## Abstract

How chromatin dynamics relate to transcriptional activity remains poorly understood. Using single-molecule tracking, coupled with machine learning, we show that histone H2B and multiple chromatin-bound transcriptional regulators display two distinct low-mobility states. Ligand activation results in a marked increase in the propensity of steroid receptors to bind in the lowest-mobility state. Mutational analysis revealed that interactions with chromatin in the lowest-mobility state require an intact DNA binding domain and oligomerization domains. These states are not spatially separated as previously believed, but individual H2B and bound-TF molecules can dynamically switch between them on time scales of seconds. Single bound-TF molecules with different mobilities exhibit different dwell time distributions, suggesting that the mobility of TFs is intimately coupled with their binding dynamics. Together, our results identify two unique and distinct low-mobility states that appear to represent common pathways for transcription activation in mammalian cells.

## Teaser

Spatial mobility of chromatin-bound transcriptional regulators is intimately coupled with their dwell times and function.

## INTRODUCTION

The eukaryotic genome is highly organized across several length scales. DNA wraps around nucleosomes to form chromatin, which loops, forms topologically associating domains (TADs), and is hierarchically packaged into chromosomes and chromosome territories (*1*). This organization is crucial for the regulation of gene expression. Genes that are present in more accessible regions or within three-dimensional (3D) proximity of their cis-acting elements (enhancers, promoters) are more likely to be expressed (*2*). Transcription factors (TFs) bind consensus motifs within enhancers and promoter-proximal regions, and this binding triggers the recruitment of cofactors, remodelers, co-repressors, or coactivators, all of which act in concert to regulate target genes (*3*). This is a highly dynamic process with TFs only transiently interacting with chromatin on a time scale of seconds (*4*–*6*). Chromatin itself is a highly dynamic polymer, subject to thermal fluctuations and active forces such as transcription (*7*), loop extrusion (*8*), DNA damage repair, and replication (*9*). How TFs navigate this complex nuclear microenvironment to find their binding sites remains poorly understood.

Over the past decade, single-molecule tracking (SMT) has emerged as a powerful tool to interrogate the dynamics of proteins in living cells. In bacteria, TFs have been shown to undergo a combination of 3D diffusion and 1D facilitated diffusion (sliding) to find their target sites (*10*, *11*). Eukaryotic nuclei present a much bigger challenge to the TF search for relevant motifs since the nucleus contains several levels of organization. Chromatin in eukaryotic cells exhibits complex dynamic signatures, showing micrometer-scale coherence on a time scale of 10 s (*12*), and recent SMT studies have found that transcription (*13*) and loop extrusion (*14*) constrain chromatin mobility. Classification of fast TF and histone H2B trajectories into five mobility groups revealed a spatial patterning of mobility states (*15*), with lower-mobility states occupying the nuclear periphery and perinucleolar regions, which are typically associated with heterochromatin. Similarly, fast SMT showed that nucleosomes exhibit two mobility states on a time scale of 500 ms, which were believed to represent spatially separate domains of “fast” and “slow” chromatin, i.e., discrete spatial domains that have different mobility states (*16*). In both these studies, single-molecule trajectories were sampled rapidly [100 Hz (*15*) or 20 Hz (*16*)] and for short times (≤500 ms). However, TF dwell times have been shown to obey power-law distributions, with some binding events lasting for tens of seconds (*17*). To identify mobility states that are important on these time scales, it is essential to study the molecules that remain bound for similar times. Furthermore, chromatin is a viscoelastic polymer showing different dynamic signatures at short and long time scales (*12*, *18*). This makes it important to complement these fast SMT studies with those sampling longer TF binding events to get a more complete picture of chromatin and TF dynamics.

Despite extensive studies of chromatin dynamics, several questions remain open: Which modes of chromatin mobility can we detect at time scales meaningful for TF binding? Are these mobility states spatially separate or can individual nucleosomes switch between them? Do TFs and co-regulators preferentially associate with a subset of chromatin mobility states? For inducible TFs, how do these associations change upon ligand activation? Which domains of TFs are key determinants of mobility and chromatin interactions?

Here, we use SMT along with a systems-level machine learning algorithm to address these questions. First, we focus on H2B as a marker for chromatin and find that chromatin exhibits two distinct low-mobility states. Individual H2B molecules dynamically switch between these states, challenging the view that chromatin forms long-lasting and spatially separated mobility domains (*19*). Next, we used our analysis framework to study steroid receptors (SRs), which are hormone-inducible TFs. We find that SRs, along with other co-regulators, show the same two low-mobility states as H2B, indicating that, on these imaging time scales, TF mobility arises from the underlying mobility of chromatin. Similar to H2B, chromatin-bound TFs and co-regulators can also switch between these two states. Upon activation of SRs, the bound fraction and the proportion of molecules in the lowest-mobility state increase substantially, indicating that binding in this state is associated with the active form of SRs. Focusing on the peroxisome proliferator–activated receptor gamma 2 (PPARγ2), we show that engagement with chromatin in the lowest-mobility state requires an intact DNA binding domain (DBD) and domains important for the formation of heterodimeric protein complexes that enhance chromatin binding and transcriptional output. We show that trajectories with distinct overall mobilities exhibit different switching characteristics and dwell time distributions. Last, we discuss our results in the context of recent studies and propose an alternative model for TF dynamics in eukaryotic cells.

## RESULTS

### Chromatin mobility is characterized by dynamic switching between two distinct low-mobility states

**Fig. 1.**
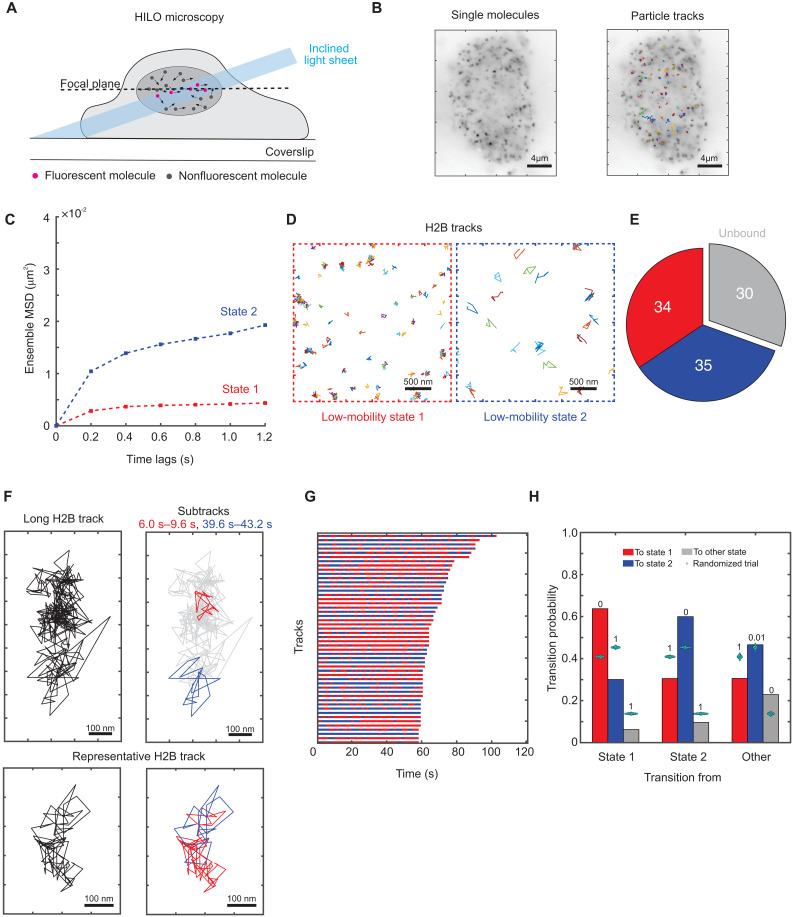
Histone H2B shows two distinct low-mobility states. (**A**) Schematic of SMT experiment. (**B**) Left: Time projection of a representative H2B-Halo SMT movie (right) overlaid with tracks. (**C**) Ensemble MSD for histone H2B (*N*_cells_ = 149, *N*_tracks_ = 25,298, and *N*_sub-tracks_ = 88,934). Error bars denote SEMs. (**D**) Sample tracks assigned to low-mobility state 1 (red) and low-mobility state 2 (blue) for H2B. (**E**) Pie chart of proportions of H2B sub-tracks assigned to different mobility states. (**F**) Top left: Representative long H2B track. Top right: Sub-tracks of length 3.6 s color-coded by state assignment (state 1 in red and state 2 in blue) to illustrate differences between the spatial extent of the two states. Bottom left: A representative 16 s H2B track. Bottom right: The same track with the sub-tracks color-coded by state assignment, showing spatial overlap between the two low-mobility states. (**G**) Temporal reconstruction of the 50 longest tracks for histone H2B. (**H**) Transition probabilities for H2B among states 1 and 2 and all other states. Cyan swarm charts represent transition probabilities calculated from 1000 randomized ensembles. The numbers above the bars represent the proportion of randomized trials that have a higher transition probability than the respective calculated transition probability.

We performed SMT on adenocarcinoma 3617 cells (*20*) expressing HaloTag-protein chimeras (with H2B serving as a probe for chromatin) to determine the spatial mobility of proteins. We labeled the HaloTag-protein chimeras with low concentrations (5 nM) of JF_549_ dye (*21*) and imaged cell nuclei using highly inclined laminated optical (HILO) sheet microscopy (*22*) (Fig. 1A). We are most interested in the spatial mobility of bound events that last on the order of tens of seconds, as they were shown to be correlated with transcriptional outcomes (*23*). Because photobleaching prevents rapid imaging for long times (*24*), we imaged the cells every 200 ms, with short exposure times of 10 ms to minimize motion blur (movie S1). Note that at these frame rates, fast diffusing molecules will rapidly disappear from the focal plane (*25*). We estimated the probability of a freely diffusing molecule to remain within our focal volume within our sampling interval of 200 ms to be <10^−9^ (see Materials and Methods). Other studies have extensively characterized these fast diffusive states for TFs while accounting for defocalization (*25*–*27*). In this study, single molecules were tracked using a custom algorithm (see Materials and Methods), and we performed various quality controls to ensure that tracking errors were minimized (see Materials and Methods, figs. S1 and S2, and table S1). By interspersing short (10 ms) illumination pulses with long (200 ms) dark intervals, we can obtain longer trajectories, with a small fraction lasting up to 2 min (fig. S3). A representative temporal projection of an H2B SMT image stack along with particle tracks is shown in Fig. 1B.

To quantify and characterize the mobility of H2B, we used a systems-level classification algorithm [perturbation expectation maximization version 2 (pEMv2)] to classify H2B trajectories into different diffusive states (*28*). pEMv2 produces discrete mobility states and posterior probability distributions that maximize a defined log-likelihood function (*28*). Given a collection of trajectories without any a priori knowledge of the underlying diffusive states, pEMv2 uses machine learning along with a Bayesian information criterion (BIC) to uncover a set of diffusive states from a complex distribution of diffusivities. To minimize errors due to transitions within a track, while retaining sufficient numbers of data points for classification, we split our tracks into sub-tracks of seven frames each (fig. S4A) (*29*). Because pEMv2 is a probabilistic algorithm, we assign each sub-track to the state for which it has the highest posterior probability, filtering out sub-tracks with similar probabilities of belonging to multiple states (fig. S4, B and C; see Materials and Methods for details). After classification by pEMv2, we removed any states with a population fraction smaller than 5% (see Materials and Methods; fig. S4C). We then used the ensemble mean-squared displacement (MSD) of these states to compare diffusive states across proteins and conditions. The MSD curve serves as a good metric for the exploration size and diffusivity of an ensemble of particles (*30*). First, we compared the MSD for all (unclassified) H2B tracks with that reported in the literature. For a time lag of 0.6 s, H2B has an MSD of ~0.013 μm^2^ (fig. S2C), which is in good agreement with previous studies that report similar MSDs on a time scale of 0.5 to 0.6 s (*14*, *31*). Next, we used pEMv2 to identify discrete mobility states.

We found that the ensemble of H2B trajectories converged to seven mobility states, but the bulk of sub-tracks were classified into two states (fig. S4C) based on the posterior probability of assignment to particular states (see Materials and Methods). Inspection of the ensemble MSD for both these states (Fig. 1C; henceforth referred to as states 1 and 2), as well as randomly sampled sub-tracks (Fig. 1D) shows that state 2 has a higher exploration radius than that of state 1 and that these states are distinct. States 1 and 2 each account for ~35% of all sub-tracks while ~30% of H2B molecules are unbound (see Materials and Methods) (Fig. 1E). Our data agree with recent studies (*16*, *29*), which showed that H2B exhibits two distinct mobility states. These states were attributed to spatially separated domains of fast and slow chromatin (*16*). However, the bulk of the data in that study represents relatively short tracks that last less than 500 ms (*16*). While each of our sub-tracks is of a comparable length (1.2 s), the parent tracks are longer, with a few lasting even up to 2 min (Fig. 1, F and G, and fig. S3).

To determine whether the two mobility states correspond to spatially separated chromatin domains that persist over seconds, we analyzed the dynamics of the two low-mobility states within individual tracks. We generated a temporal reconstruction of state dynamics by coloring in sub-tracks by the color of the state they are assigned to (fig. S4, D and E). Notably, we found that the same H2B molecule dynamically switches between both low-mobility states as shown for representative tracks in Fig. 1F. Note that while we have picked spatially separated sub-tracks for ease of visualization (Fig. 1F, top), state 1 and state 2 sub-tracks overlap throughout the parent track [Fig. 1F (bottom), fig. S4D, and movies S2 to S4]. While this argues against large-scale spatial separation, our imaging modality does not have the *z*-resolution to preclude the possibility of smaller-scale spatial separation in three dimensions. More generally, across an ensemble of the 50 longest tracks, we observed similar switching behavior between these two states (Fig. 1G).

We then quantified the transition probabilities for all tracks that contain at least three sub-tracks (see Materials and Methods). To determine whether these transition probabilities are statistically significant, we performed a permutation test: We generated 1000 ensembles of randomly permuted sub-tracks and calculated the transition probabilities for these ensembles (see Materials and Methods). This approach has been used previously to test the statistical significance of transition matrices in atmospheric Markov chains (*32*). Our analysis shows that H2B molecules in states 1 and 2 prefer to remain in the same state (Fig. 1H) with a higher probability than would be expected from random ensembles with the same population fractions (Fig. 1H). On the other hand, while we do observe transitions from state 1 to state 2 and vice versa, our permutation test shows that these transitions occur less frequently than in a random ensemble (Fig. 1H).

Together, our data suggest that rather than forming large-scale spatially separated domains of higher or lower mobility, chromatin can switch dynamically between these two mobility states. However, this only becomes apparent when we track nucleosomes over longer time scales.

### SRs exhibit the same two low-mobility states as H2B, with ligand-dependent population fractions

Having established that H2B has two dynamic mobility states, we turned our attention to TFs. How is TF mobility different from that of H2B? Do active and inactive forms of a TF behave differently? To answer these questions, we applied our analysis framework to study multiple SRs, which are class I nuclear receptors that bind hormone response elements (HREs) as homodimers or homotetramers (*33*, *34*). Some SRs, such as the glucocorticoid receptor (GR) and the androgen receptor (AR), are predominantly cytoplasmic in the absence of hormone with a small nuclear fraction, while the estrogen receptor (ER) is mostly nuclear (*35*). In the case of the progesterone receptor (PR), it can be either predominantly cytoplasmic or nuclear, depending on the isoform (*36*). Agonist binding triggers a conformational change, nuclear translocation (for GR, AR, and PR), oligomerization, and binding to HREs. We tracked unliganded ER and the small nuclear fraction of unliganded GR, PR, and AR and contrasted these with their corresponding ligand-activated receptors.

**Fig. 2.**
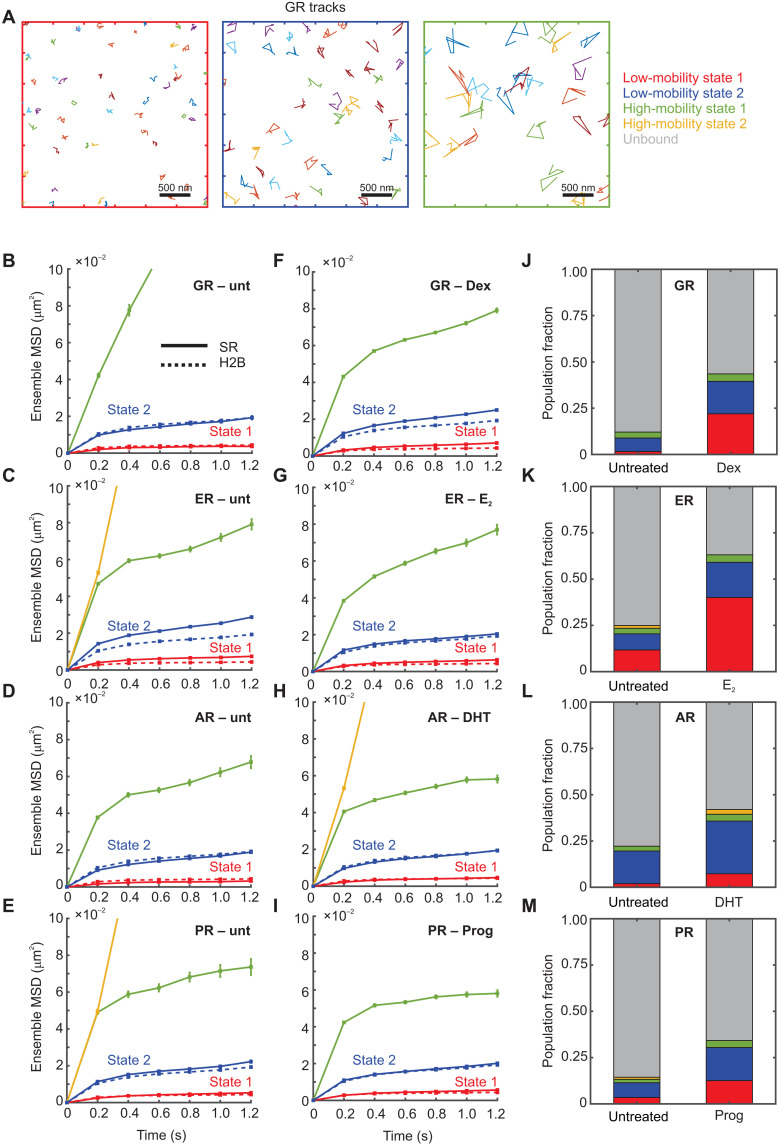
Steroid receptors also exhibit two low-mobility states, with ligand-dependent population fractions. (**A**) Sample tracks for the GR. Left: Low-mobility state 1. Middle: Low-mobility state 2. Right: High-mobility state. (**B** to **I**) Ensemble MSD for indicated steroid receptor (solid lines) and histone H2B (dashed lines). Error bars denote SEMs. (B) Untreated GR (*N*_cells_ = 35, *N*_tracks_ = 386, and *N*_sub-tracks_ = 962). (C) Untreated ER (*N*_cells_ = 49, *N*_tracks_ = 4057, and *N*_sub-tracks_ = 9551). (D) Untreated AR (*N*_cells_ = 51, *N*_tracks_ = 1394, and *N*_sub-tracks_ = 4001). (E) Untreated PR (*N*_cells_ = 37, *N*_tracks_ = 1371, and *N*_sub-tracks_ = 3197). (F) GR activated with dexamethasone (Dex) (*N*_cells_ = 238, *N*_tracks_ = 30,652, and *N*_sub-tracks_ = 81,172). (G) ER activated with 17β-estradiol (E_2_) (*N*_cells_ = 50, *N*_tracks_ = 8147, and *N*_sub-tracks_ = 24,299). (H) AR activated with dihydrotestosterone (DHT) (*N*_cells_ = 38, *N*_tracks_ = 5160, and *N*_sub-tracks_ = 12,697). (I) PR activated with progesterone (Prog) (*N*_cells_ = 41, *N*_tracks_ = 4951, and *N*_sub-tracks_ = 14,899). (**J** to **M**) Comparative bar charts showing population fractions of various states for the indicated steroid receptors.

All tested SRs, with and without activation by hormone, exhibit two distinct low-mobility states and a small population of one or two higher-mobility states (Fig. 2, A to I, and fig. S5). Because of our slow imaging rate, any fast-diffusing molecules will rapidly move away from the focal plane (*25*). Because a majority of the sub-tracks belong to the two low-mobility states (fig. S5, A to H), we focus on these for the rest of the study. As can be seen qualitatively from sample tracks belonging to these states (Fig. 2A) and quantitatively from ensemble MSD plots (Fig. 2, B to I), these states have different mobility signatures. On comparing these with the states recovered for H2B, we find that all the examined SRs exhibit the same two low-mobility states as H2B on our observation time scales (Fig. 2, B to I).

To better understand the biological origin of these mobility states for SRs, we compared the population fractions of the states before and after hormone activation. All four SRs show an increase in the overall bound fraction upon activation (Fig. 2, J to M). All SRs show a marked increase in the proportion of the lowest-mobility state (state 1): 10-fold for GR (Fig. 2J), 3.3-fold for ER (Fig. 2K), 3.5-fold for AR (Fig. 2L), and 4-fold for PR (Fig. 2M). These are accompanied by a smaller increase in the relative proportion of state 2: 2.5-fold (GR), 2.1-fold (ER), 1.5-fold (AR), and 2.2-fold (PR) (Fig. 2, J to M). Note that 3617 cells do not express endogenous AR and PR (*37*, *38*) and therefore may not provide a native chromatin context for AR and PR binding. This likely results in the relatively low population fractions observed for state 1 in these cells (Fig. 2, L and M). As noted above, GR, AR, and PR are primarily cytoplasmic in the absence of hormone. Thus, the unliganded GR, AR, and PR tracks come from the small nuclear fraction of the receptor, which results in substantially fewer tracks per cell as compared to their liganded counterparts (fig. S1, C to J). Accurate estimation of the unbound fraction will require faster imaging rates and will likely result in an even smaller bound fraction, which will exaggerate any differences in the population fraction of liganded versus unliganded GR, AR, and PR. As ER is always nuclear, we can track a similar number of molecules in both unliganded and liganded states and found that ER exhibits similar changes in state 1 proportions upon activation as the other SRs. Together, these data suggest that the tendency for SRs to bind in state 1, the lowest chromatin mobility state, better correlates with their activation status than does state 2, which implies that either binding of activated SR to chromatin constrains its mobility and/or activated SRs are more capable of interacting with chromatin in state 1.

We used two alternative methods to test the generality of our observed mobility states. Given a collection of trajectories, we can calculate the self part of the van Hove correlation (vHc) function or step-size distribution. The empirical vHc can then be approximated as a superposition of Gaussian basis functions (see Materials and Methods), from which we can iteratively calculate the distribution of MSDs that gives rise to the calculated vHc. We used the iterative algorithm developed by Richardson (*39*) and Lucy (*40*) and successfully implemented it to study nucleosome dynamics (*16*) and to calculate the distribution of MSD (or, equivalently, the diffusivity distribution). We refer to this analysis as “RL analysis” in the rest of the manuscript. We find once again that the predicted MSD distribution for H2B, shown here for a time lag of 0.8 s (fig. S6A) has two main populations, confirming the two states recovered from pEMv2. The bimodal distribution of MSDs was observed for other time lags (0.6 and 1.0 s) as well (fig. S6), indicating the generality of our findings. A similar analysis for hormone-activated SRs (GR, ER, AR, and PR) also showed two distinct low-mobility states supporting our pEMv2 results (fig. S6). Consistent with the thinly populated higher-mobility states detected by pEM, we observe some higher-mobility states in the distribution of MSDs as well (fig. S6).

Fitting displacement histograms to diffusion models has been used to estimate multiple mobility states within an ensemble of trajectories (*25*, *31*). However, this approach requires a priori knowledge of the number of diffusive states and assumes that displacements are independent (Markovian dynamics), which, therefore, can only account for normal diffusion. The viscoelastic nature of chromatin as well as static and dynamic localization noise may render the protein displacements non-Markovian (*41*). However, pEMv2 accounts for these constraints (*11*, *12*). We then asked whether the states identified by pEMv2 are consistent with multistate diffusion models. We sorted the sub-tracks into the two states defined by pEMv2 and fit displacement histograms of these ensembles of sub-tracks to a two-state model using Spot-On (*25*). Fitting to a three-state model resulted in only two distinct states. We compared the Spot-On diffusion coefficients and localization errors with those obtained from pEMv2 (fig. S6, P and Q). We find a strong correlation between the diffusion constants recovered from both methods (*R*^2^ = 0.93764, correlation coefficient = 0.9683; fig. S6P). We then compared the localization error calculated by pEMv2 from all the input tracks to those recovered from Spot-On. We find that the localization errors also show a very strong correlation (*R*^2^ = 0.98467, correlation coefficient = 0.9923; fig. S6Q). In conclusion, we find that all tested methods converged on the detection of two low-mobility states.

### SRs dynamically switch between the two low-mobility chromatin states

**Fig. 3.**
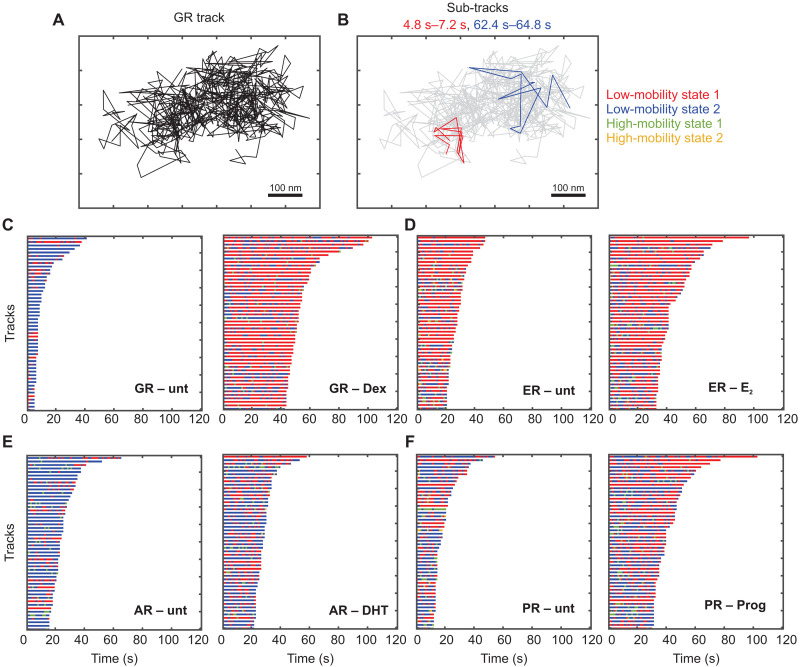
TFs dynamically switch between two low-mobility states. (**A**) Sample GR track (same as the track shown in fig. S4D). (**B**) Sub-tracks of length 2.4 s from the same track as in (A) are color-coded by state assignment (state 1 in red and state 2 in blue). (**C** to **F**) Temporal reconstruction for the 50 longest tracks for steroid receptors. Left: Without hormone. Right: Upon activation by hormone. (C) GR. (D) ER. (E) AR. (F) PR.

Because we observe H2B molecules switching between the two low-mobility states, we next examined whether SRs also exhibited similar switching behaviors. Visual inspection of tracks showed that the same GR molecule could switch between these two mobility states (Fig. 3, A and B), with the state 2 sub-tracks exhibiting larger jumps (Fig. 3B). We then compared the switching behavior of SRs before and after hormone stimulation.

Quantifying the probability of transitions between these states, we observed that GR (fig. S7, A and B), ER (fig. S7, C and D), and PR (fig. S7, E and F) molecules in states 1 and 2 prefer to remain in the same state, while transitions into state 2 are dominant for AR (fig. S7, G and H). Ligand activation results in an ~13% increase in state 1 to state 1 transitions for GR and a corresponding 6% increase for ER (fig. S7, A to D), while AR and PR show very subtle differences with and without agonist (fig. S7, E to H). For unliganded SRs, state 2 to state 1 transitions are not significantly different at the 99% confidence level from those obtained for ensembles of random permutations (fig. S7, A, C, E, and G; see Materials and Methods). However, upon ligand activation, these transitions occur with a higher probability than corresponding transitions for unliganded SRs (fig. S7) but occur less frequently than the random ensemble. These data suggest that activation of SRs by hormone results in an increase in transitions into state 1 and that these transition probabilities are significantly different from those in a random ensemble.

Collectively, our data show that SRs bind to both low-mobility chromatin states. Furthermore, SRs frequently switch between these chromatin states, underscoring the fact that these states are not spatially separated. Ligand activation markedly increases the population fraction of SRs in state 1, implying that the propensity of SRs to bind to state 1 chromatin increases substantially upon hormone activation. The remarkable similarities between TFs and H2B dynamics led us to propose that the TFs, by virtue of being bound to chromatin, exhibit the same mobility states as chromatin.

### Other transcriptional regulators also exhibit two distinct mobility states

Because we observed two distinct mobility states for SRs, which represent SRs bound to chromatin exhibiting different mobility states, we hypothesized that other transcriptional regulators should also exhibit these two states. To test this hypothesis, we performed SMT experiments and subsequent analysis on several nuclear proteins that bind DNA or chromatin and perform different functions.

RELA/p65 is an important subunit of the nuclear factor κB (NF-κB) TF, which is activated in response to many external stimuli (*42*). GR-interacting protein 1 (GRIP1), also known as nuclear receptor coactivator 2 (NCoA2) is a co-regulatory protein that is recruited to DNA by nuclear receptors in response to ligand-activation (*43*). GRIP1 facilitates nuclear receptor–mediated gene regulation by acetylating histone tails, thereby modulating chromatin accessibility (*43*). Mediator of RNA polymerase II transcription subunit 26 (MED26) is a subunit of the mediator complex that assists RNA polymerase II (RNA Pol II)–mediated transcription by recruiting accessory proteins that promote transcriptional elongation (*44*). SWI/SNF-related, matrix-associated, actin-dependent regulator of chromatin, subfamily A, member 4 (SMARCA4, also known as BRG1) is an adenosine triphosphate–dependent remodeler that is a part of the SWI/SNF complex. SMARCA4 modulates gene expression by changing chromatin accessibility through its remodeling activity (*45*). CCCTC-binding factor (CTCF) is important for 3D genome organization, leading to the formation of enhancer-promoter loops and regulating the structure of TADs (*1*). Many of these regulators have been the focus of previous SMT studies—RELA (*46*), GRIP1 (*24*), SMARCA4 (*17*, *24*), and CTCF (*17*, *26*, *47*)—but with a focus on either measuring dwell times (*17*, *24*, *46*, *47*) or fast diffusing molecules (*26*).

**Fig. 4.**
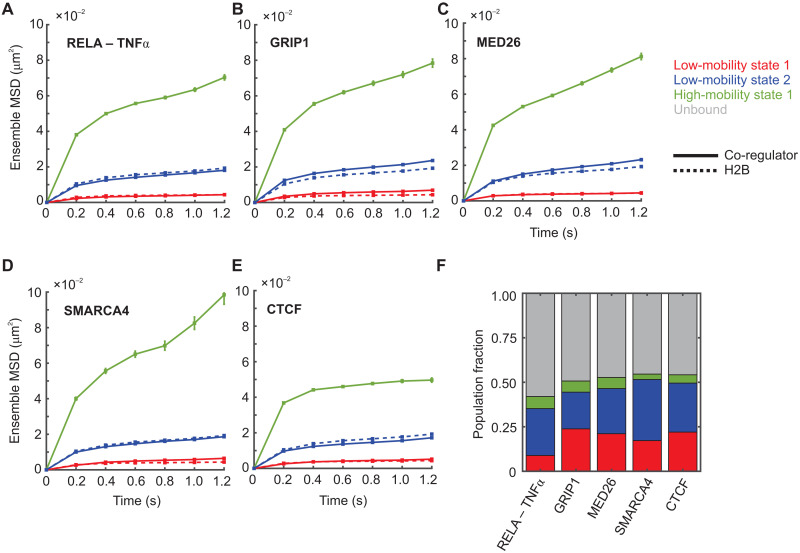
Other transcriptional regulators also exhibit two distinct low-mobility states. (**A** to **E**) Ensemble MSD plots for the indicated transcriptional co-regulator (solid lines) and histone H2B (dashed lines). Error bars denote SEMs. (A) RELA/p65 activated with TNFα (*N*_cells_ = 67, *N*_tracks_ = 9524, and *N*_sub-tracks_ = 24,634). (B) GRIP1 (*N*_cells_ = 36, *N*_tracks_ = 4847, and *N*_sub-tracks_ = 14,010). (C) MED26 (*N*_cells_ = 57, *N*_tracks_ = 11,429, and *N*_sub-tracks_ = 29,085). (D) BRG1/SMARCA4 (*N*_cells_ = 22, *N*_tracks_ = 3179, and *N*_sub-tracks_ = 8112). (E) CTCF (*N*_cells_ = 69, *N*_tracks_ = 10,457, and *N*_sub-tracks_ = 34,503). (F) Comparative bar chart showing population fractions for the indicated co-regulators.

For this diverse set of transcriptional proteins with widely varying functions, we observed two qualitatively similar low-mobility states as histone H2B (Fig. 4 and fig. S8). As seen with SRs, these transcriptional regulators also switch between the two low-mobility states (fig. S9), with molecules preferentially remaining in the same state (fig. S9), except RELA and SMARCA4, which show a slight preference to switch from state 1 to state 2 [fig. S9, A and D (right)]. These data suggest that all detected TF and co-regulator dynamics reflect the mobility of the local chromatin environment.

### PPARγ2 requires an intact DNA binding and oligomerization domain to bind to state 1 chromatin

**Fig. 5.**
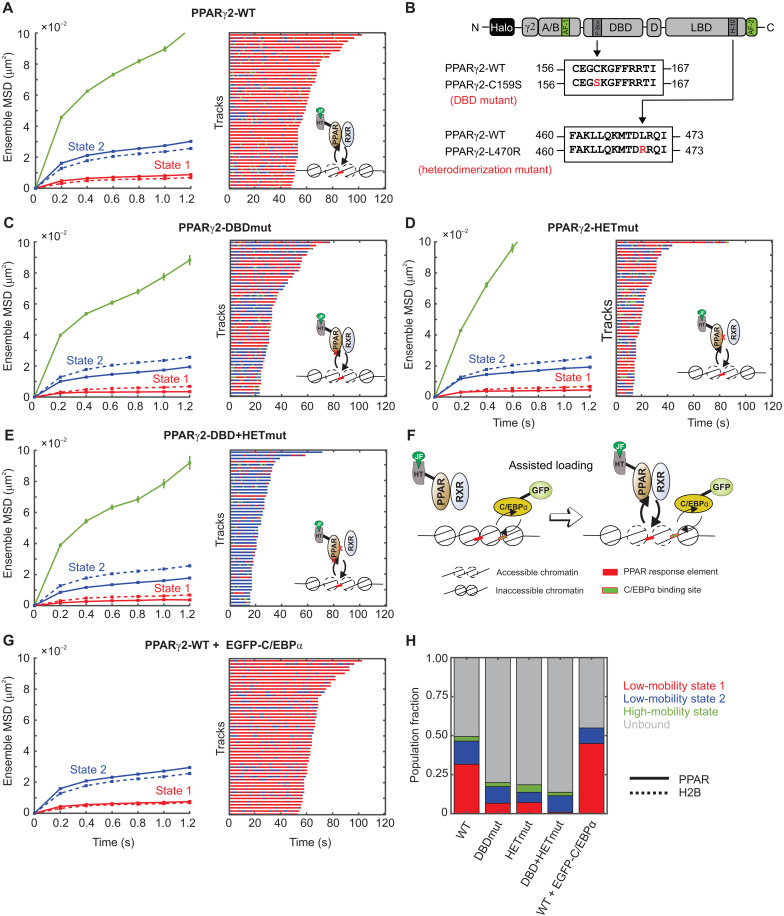
State 1 for PPARγ2 requires intact DBD and the ability to form heterodimeric complexes. (**A**) Left: Ensemble MSD of H2B (dashed lines, *N*_cell_ = 54, *N*_tracks_ = 8522, and *N*_sub-tracks_ = 29,262) and wild-type PPARγ2 (solid lines, *N*_cells_ = 127, *N*_tracks_ = 20,983, and *N*_sub-tracks_ = 62,848). Error bars indicate SEMs. Right: Temporal reconstruction of the 50 longest tracks along with (inset) a cartoon depicting PPARγ2 binding to PPAR response elements (PPRE). (**B**) Schematic of point mutations to abrogate the DBD and heterodimerization domains of PPARγ2. (**C** to **E**) Left: Ensemble MSD for indicated PPARγ2 mutant. Error bars denote SEMs. Right: Temporal reconstruction of the 50 longest tracks colored by state assignment: (C) PPARγ2-DBD mutant (PPARγ2-DBDmut) (*N*_cells_ = 38, *N*_tracks_ = 3721, and *N*_sub-tracks_ = 9872), (D) PPARγ2-heterodimerization mutant (PPARγ2-HETmut) (*N*_cells_ = 28, *N*_tracks_ = 1728, and *N*_sub-tracks_ = 4049), and (E) PPARγ2-DBD + HET mutant (*N*_cells_ = 46, *N*_tracks_ = 1695, and *N*_sub-tracks_ = 4046). (**F**) Assisted loading model for C/EBPα-mediated PPARγ2 loading. (**G**) Left: Ensemble MSD for PPARγ2-WT with overexpression of GFP-C/EBPα (*N*_cells_ = 89, *N*_tracks_ = 18,912, and *N*_sub-tracks_ = 63,842). Error bars denote SEMs. Right: Temporal reconstruction of the 50 longest tracks. (**H**) Comparative population fractions for all PPARγ2 variants.

To understand the factors that determine the partitioning of TFs into the two mobility states, we focused on PPARγ2, which is a class II nuclear receptor that binds chromatin as a heterodimer with the retinoid X receptor [RXR; Fig. 5A, right (inset)] (*48*). In particular, the existence of well-characterized interacting partners and DNA binding and heterodimerization mutants allows for a systematic study of PPARγ2’s mobility states. We chose 3T3-L1 mouse pre-adipocytes as our model cell line to study PPARγ2 because PPARγ2 is functionally important for adipogenesis (*49*, *50*). This allows us to study a TF with functional relevance in its native chromatin context.

PPARγ2 is one of two PPARγ isoforms expressed from the *PPARG* gene. PPARγ2 contains 30 additional amino acids on its N-terminal end as compared to PPARγ1 (Fig. 5B). PPARγ1 is expressed in almost all tissues, but PPARγ2 is predominantly found in adipose tissue and is important for adipocyte differentiation, fatty acid storage, and glucose metabolism and is a known therapeutic target for diabetes (*51*, *52*). During adipogenesis, PPARγ2 and CCAAT enhancer-binding protein α (C/EBPα) act in concert to regulate genes essential for this process (*53*).

We first transiently expressed HaloTag-fused H2B and PPARγ2 chimeras in 3T3-L1 cells, performed SMT, and analyzed the data with the above-described workflow. As observed in 3617 cells, PPARγ2 and H2B exhibit the same two low-mobility states (Fig. 5A, left). Both PPARγ2 and H2B in 3T3-L1 cells exhibit switching between the two lowest-mobility chromatin states as seen for other chromatin-bound TFs and H2B [Fig. 5A (right) and fig. S10, A and B]. While H2B molecules in both state 1 and state 2 preferentially transition to the same state (fig. S10A), PPARγ2 molecules in state 1 remain in state 1 ~70% of the time but show an equal transition probability from state 2 into both states 1 and 2 (fig. S10B).

To test the role of the DBD and the heterodimerization domain (HET) in the two low-mobility states, we first mutated the 159th amino acid from cysteine to serine (C159S; henceforth referred to as DBDmut), which has been shown to disrupt the zinc finger and prevent sequence-specific chromatin interactions in vitro, and abrogate transcriptional responses [Fig. 5, B and C (right, inset)] (*54*). Disruption of the DBD results in a marked reduction in the overall bound fraction and, particularly, the population fraction of state 1 as compared to that of wild-type PPARγ2 (PPARγ2-WT) (Fig. 5, C and H). However, we do not completely lose the bound fraction or the binding in state 1 (Fig. 5, C and H). This is consistent with previous studies that showed that RXR binding to the 3′ half-site of PPAR response elements is more important than PPARγ2 binding to the 5′ half-site for the PPARγ2:RXR complex to stabilize engagement with chromatin (*54*). We also observed an ~18% increase in the probability of state 2 molecules to remain in state 2, along with a concomitant decrease of ~19% in the state 2 to state 1 transition probability (fig. S10, B and C). This suggests that the DBD is important for PPARγ2 to transition from state 2 to state 1.

Mutation of the 470th amino acid from leucine to arginine (L470R; henceforth referred to as HETmut) eliminates the heterodimerization interface with RXR and has been shown to be transcriptionally inactive [Fig. 5, B and D (right, inset)] (*55*, *56*). We analyzed this construct to find very similar results to those obtained for the DBD mutant. The overall bound fraction was much smaller than that of PPARγ2-WT, but the same as that of PPARγ2-DBDmut (Fig. 5H). The relative proportion of state 1 was also similar to that of PPARγ2-DBDmut (7%), indicating that monomeric PPARγ2 is still capable of interacting with chromatin, potentially through its intact DBD (Fig. 5H).

By introducing both the DBD and HET mutations simultaneously [PPARγ2-DBD + HETmut; Fig. 5, B and E (right, inset)], we observed that the PPARγ2-DBD + HETmut has an even smaller bound fraction and a vanishingly small proportion of state 1 as compared to those for PPARγ2-WT (Fig. 5, E and H). While canonical models of TF-chromatin interactions focus on the binding of a factor through the DBD, recent studies have implicated intrinsically disordered regions (IDRs) within a TF in directing binding specificity near cognate binding sites (*57*, *58*). PPARγ2-DBD + HETmut still exhibits binding in state 2, which could result from chromatin interactions through IDRs.

Similar to PPARγ2-DBDmut, PPARγ2-HETmut has an impaired ability to transition from state 2 to state 1 (fig. S10D). As compared to PPARγ2-WT, PPARγ2-DBD + HETmut shows a 31 to 40% decrease in transitions into state 1 and a 34 to 38% increase in transitions into state 2 (fig. S10E). PPARγ2-DBD + HETmut molecules preferentially switch to state 2 from all states (fig. S10E). Because we have seen that an increase in the proportion of state 1 along with increased transitions into state 1 (from both states 1 and 2) are associated with the active form of SRs (Figs. 2 and 3, and fig. S7), these data also support the hypothesis that TF engagement with chromatin in state 1 correlates with transcriptional activity. Because these mutations reduce the ability of PPARγ2 to interact with chromatin, we also tested the opposite perturbation: What happens to the two states if we facilitate PPARγ2 binding?

C/EBPα and PPARγ2 have been shown to participate in dynamic assisted loading at closed chromatin sites by recruiting remodelers (Fig. 5F) (*50*). To further test our hypothesis, we overexpressed enhanced green fluorescent protein (EGFP)–fused C/EBPα, which should promote PPARγ2-chromatin interactions in state 1 (Fig. 5F). Consistent with our hypothesis, overexpression of C/EBPα resulted in an increase in the overall bound fraction of PPARγ2 (Fig. 5H) and a 1.4-fold increase in the proportion of state 1 (Fig. 5H). In contrast to the PPARγ2-DBD + HETmut data, overexpression of EGFP-C/EBPα results in a 9 to 16% increase in transitions into state 1 along with an 11 to 17% decrease in transitions to state 2, with all states showing a preference to switch to state 1 (fig. S10F). Together, our data indicate that binding in state 1 requires an intact DBD and heterodimerization domain and that active TFs show a higher proclivity for this state than do inactive TFs.

### Tracks with different exploration radii exhibit different switching characteristics and dwell times

After analyzing sub-tracks using pEMv2, we found that all tested molecules dynamically switch between two low-mobility states. We then used the Richardson-Lucy (RL) analysis to confirm that two states can be recovered from the calculated vHc function (fig. S6). Since the RL analysis produces a distribution of MSDs, we can use the minima in the MSD distribution to classify full-length tracks, rather than sub-tracks, into lower (henceforth referred to as RL group 1) or higher mobility (henceforth referred to as RL group 2) populations (fig. S11, A to C). We calculate the MSD for each track at a time lag of 0.8 s and classify the track into RL group 1 or 2 as shown in fig. S11 (A to C). Because these tracks consist of sub-tracks previously analyzed by pEMv2, we can examine the pEMv2 population fractions and the state-switching characteristics within each RL group.

**Fig. 6.**
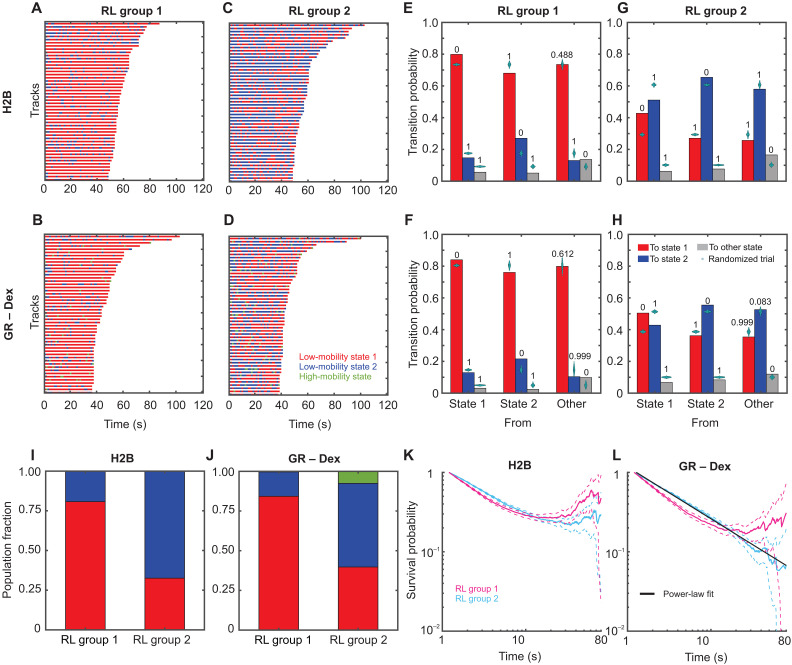
Tracks with different exploration radii exhibit distinct switching patterns. (**A** to **D**) Temporal reconstruction of the 50 longest tracks of single molecules belonging to RL group 1 (overall lower mobility) (A and B) and to RL group 2 (overall higher mobility) (C) and (D). The tracks are color-coded to show the pEM-identified states of the 1.2-s segments making up the entire track. State 1 is depicted in red, and state 2 is in blue. Higher-mobility states are colored green and yellow: (A) and (C) H2B. (B) and (D) GR activated with Dex. (**E** to **H**) Transition probabilities calculated for molecules in RL group 1 (E) and (F) and RL group 2 (G) and (H). Transitions into pEM state 1 are shown in red; those into state 2 are shown in blue, and others are in gray. Cyan swarm charts show the results of the transition probability calculation for 1000 randomly permuted ensembles. Numbers above the bars display the proportion of these trials with a transition probability higher than the respective calculated transition probability: (E) and (G) H2B and (F) and (H) GR activated with Dex. (**I** and **J**) Fraction of tracks in pEM state 1 (red), pEM state 2 (blue), and pEM state 3 (green) for trajectories classified into RL groups 1 and 2 for H2B (I) and GR activated with Dex (J). (**K** and **L**) Photobleaching corrected survival probability distributions of dwell times for tracks classified into RL group 1 (magenta) and RL group 2 (cyan). Dashed lines indicate the 99% confidence interval. (K) H2B and (L) GR activated with Dex. The black line in (L) represents a power-law fit to the survival distribution for RL group 2 with an exponent β = −0.65 ± 0.01.

Analysis of these populations revealed that molecules belonging to RL group 1 (i.e., with MSD at 0.8-s time lag lower than 0.0075 μm^2^) were predominantly in state 1 (Fig. 6, A and B, and fig. S11D). Molecules belonging to RL group 2 (i.e., with MSD at 0.8-s time lag between 0.0075 and 0.028 μm^2^) exhibited appreciable fractions of both state 1 and state 2 (Fig. 6, C and D, and fig. S11E). Molecules in RL group 1 preferentially transition to state 1 (Fig. 6, E and F, and fig. S11F), while those in RL group 2 exhibit a significantly higher probability of switching between these two states (Fig. 6, G and H, and fig. S11G). This can also be seen by comparing the population fractions of the different mobility states within the two groups (Fig. 6, I and J, and fig. S11H).

Classification of tracks into RL groups allows us to calculate the survival distribution (dwell times) of H2B and GR within each of these groups. The dwell times measure the length of time that a molecule remains within the focal volume before being lost because of photobleaching or unbinding and diffusing out (*59*). First, by fitting the H2B survival distribution of all tracks to a triple exponential distribution, we estimated the photobleaching time constant (see Materials and Methods) as previously described (*17*). We then classified the H2B tracks into the two RL groups and calculated the photobleaching-corrected survival distribution of each of the groups. We find that tracks in both RL groups show similar survival distributions, with both distributions showing a plateau at longer times, as expected for long-lived molecules (Fig. 6K) (*17*). The same analysis on GR tracks shows that while RL group 1 plateaus at long times (like H2B), RL group 2 exhibits a power-law survival distribution with an exponent of β = −0.65 ± 0.01 (Fig. 6L). Therefore, TF molecules that are bound to less mobile chromatin show limited switching and histone-like dwell times. However, TFs bound to more dynamic chromatin (with overall higher MSDs and more frequent transitions between the mobility states) show similar mobility transitions but are not as long lived (exhibiting power-law dwell times; Fig. 6L). In this sense, while the switching is reflective of the dynamics of the underlying chromatin mobility, the overall dwell times are reflective of the binding, which can either be similar to histones (tightly bound) or power-law (sampling a broad range of affinities). Thus, the duration of time spent in either of the mobility states can be exponentially distributed, but the overall bound time can be power-law distributed (*17*). Combining track-level and sub-track–level analyses thus provides a powerful tool to distinguish between persistent and transient engagement with state 1.

## DISCUSSION

SMT is a powerful technique to study the intranuclear dynamics of individual proteins at the nanoscale with high temporal resolution. Here, using SMT along with a machine learning–based classification algorithm, we identify two distinct low-mobility states for histone H2B (Fig. 1). Previous studies have also found multiple mobility states for H2B (*15*, *16*). However, unlike that in (*15*), our model is not constrained to a fixed number of states. We allow our algorithm to explore up to 15 different states and find that only two states meet our statistical criteria (fig. S4C). Given the differences in imaging conditions and analysis techniques, it is not straightforward to compare our two low-mobility states with the five states described in (*15*). The spatial patterning of different mobility states was also reported with very short tracks (<500 ms) (*15*, *16*). Because chromatin is a viscoelastic polymer (*19*), the short and long time scale dynamics are likely to provide very different information. Here, we examine longer time scales on the order of tens of seconds and up to 2 min. Although we analyze 1.2 s sub-tracks using pEMv2, tracking the same molecule over longer times allows us to identify hitherto hidden transitions between the two low-mobility states. We find that unlike previous models (*16*), H2B does not form spatially separated domains of fast and slow chromatin. Instead, individual H2B molecules could dynamically switch between the two low-mobility states (Fig. 1, F to H).

We showed that multiple TFs and co-regulators switch between the same two mobility states as H2B (Figs. 2 to 5). These data indicate that presumed bound events can exhibit distinct mobility states. Using ligand-activated SRs, we determine that the active form of the TF binds more in the lowest-mobility chromatin state as compared to its inactive counterpart (Fig. 2). PPARγ2 mutants show that binding in state 1 requires an intact DBD and an RXR heterodimerization domain (Fig. 5). To confirm that this state is associated with an active TF, we showed that overexpression of EGFP-tagged C/EBPα, a TF that is known to cooperate with PPARγ2 at the chromatin level and facilitate its binding to dual target sites in chromatin (*50*), leads to an increase in the proportion of PPARγ2 molecules in state 1.

**Fig. 7.**
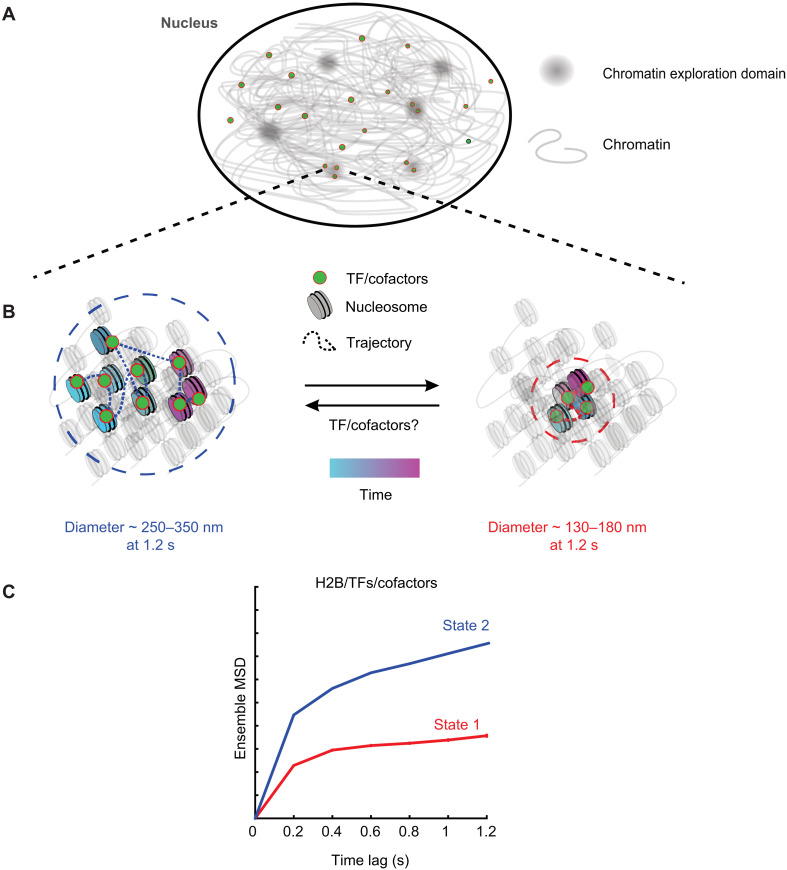
Two-state model for chromatin and transcriptional regulators. (**A**) Over short time scales (~1.2 s), chromatin mobility is constrained within chromatin exploration domains. (**B**) Within these domains, TFs/cofactors engage with chromatin, and the TF-chromatin complex can exist in one of two low-mobility states. Left: The higher of these two mobility states (state 2) has an exploration diameter of ~250 to 350 nm at 1.2 s. The trajectory shows the motion of a single TF/cofactor molecule over time. The lower-mobility state (state 1) has an exploration diameter of ~130 to 180 nm, and the motion of a single TF/cofactor molecule is represented in the right panel. Chromatin and associated TFs/cofactors can dynamically switch between these two mobility states. TF binding can promote a switch from state 2 to state 1 or unbind from state 2 chromatin and bind to state 1 chromatin within the localized chromatin domain. (**C**) The MSD plot of tracks classified by perturbation expectation maximization (pEMv2) is used to visualize the two different mobility states under the time scale of a single sub-track (1.2 s). The exploration diameter of the states is estimated as $d=2\sqrt{\mathrm{MSD}(1.2\;s)}$.

Together, our data suggest a two-state model for the mobility of chromatin wherein TFs, by virtue of being bound to chromatin, exhibit the same mobility states as chromatin. Moreover, chromatin and bound TFs can transition between these two mobility states due to processes yet to be determined. Chromatin is a viscoelastic polymer that has been shown to exhibit sub-diffusive dynamics (*18*). On our experimental time scales, chromatin explores a finite region of space that we call a chromatin exploration domain (CED; Fig. 7A). Within these CEDs, chromatin can exist in one of the two mobility states. On a time scale of 1.2 s, the lowest-mobility state has an exploration diameter of ~130 to 180 nm, while the higher-mobility state has an exploration diameter of ~250 to 350 nm (Fig. 7, B and C). Superresolution microscopy has revealed sub-TAD chromatin nanodomains (CNDs) encompassing 10 to 100 kb of DNA (*60*) and smaller nucleosome clutch domains (*61*, *62*). While it is tempting to relate our CEDs to CNDs and clutch domains, CEDs represent a temporal exploration size that depends on the time scale of interrogation. CNDs and nucleosome clutches refer to the spatial extent of nucleosomal aggregates in a static snapshot of the cell. Simultaneous tracking of nucleosomes and the CNDs/clutch domains at different time scales will help distinguish between the mobility of CNDs/clutch domains and that of their constituent nucleosomes.

Our data suggest that the observed mobility states of TFs and other transcriptional regulators result from the underlying mobility of chromatin itself. However, as shown by our results from ligand activation and mutations, the propensity of TFs to engage with one type of mobile chromatin versus another depends on their activation status and the presence of intact DBD and oligomerization domains. We also anticipate that TF binding can, in turn, influence chromatin makeup and dynamics. This process would require the TF to recruit cofactors and the transcriptional machinery, which will further depend on their activation status and the intactness of the DBD and other domains. The lack of state 1 for PPARγ2-DBD + HETmut could thus be a combination of the protein being unable to bind to chromatin in state 1 and not being able to convert state 2 chromatin to state 1 chromatin, but further work is necessary to distinguish between these possibilities.

Experimentally, whether TF binding can cause changes in chromatin mobility remains unclear. However, there is mounting experimental evidence in favor of this. RNA Pol II–mediated transcription has been shown to constrain nucleosome mobility (*7*, *13*, *63*). Consistent with this result, TF binding and subsequent recruitment of the transcriptional machinery could trigger a transition of the local chromatin polymer (and of the bound TF) from state 2 to state 1 (Fig. 7B). Similarly, loop extrusion and nucleosome-nucleosome interactions have also been shown to constrain nucleosome mobility (*14*). However, directly establishing this will require advances in imaging to allow simultaneous tracking of a TF and a specific genomic locus at high spatial and temporal resolution.

We hypothesize that any transition to state 1 is the result of a combination of processes, composed of but not limited to TF binding, RNA Pol II elongation, and loop extrusion. The following predictions emerge from this model. Inhibition of RNA Pol II with pharmacological drugs such as α-amanitin or DRB, both of which inhibit RNA Pol II elongation through different mechanisms, should result in an increase in the population fraction of state 2 and a reduction in the population fraction of state 1. Without our classification scheme, this would appear as an increase in the overall MSD of H2B, as recently reported (*13*). Similarly, rapid degradation of the RNA Pol II subunit RPB1 or the cohesin complex subunit RAD21 using the auxin-inducible degron system would also result in an increase in the population fraction of state 2 relative to that of state 1 (*13*, *14*).

Our PPARγ2 mutagenesis experiments suggest that binding in state 2 is independent of the DBD and heterodimerization domains. Because IDRs within TFs have been shown to direct TF binding independent of the DBD, we propose that regions within IDRs of TFs might be responsible for binding state 2 chromatin. However, the recruitment of cofactors is necessary for the conversion of state 2 chromatin to state 1. Targeted deletions within TF IDRs will help test this hypothesis and will be the subject of future studies.

As we have shown previously, TFs exhibit power-law distributed dwell times (*17*, *23*). This broad distribution of dwell times renders it impossible to distinguish between specific and nonspecific binding based on residence times alone. Different response elements are likely to present TFs with a broad affinity landscape. On the other hand, measuring the spatial mobility of TFs allowed us to identify two distinct mobility states across several classes of TFs in two cell lines. This opens other lines of inquiry hitherto unavailable through SMT.

By classifying H2B and GR tracks into two RL groups, we show that depending on their overall mobility, particles exhibit different binding modes, as measured by survival distributions (Fig. 6, K and L). Regardless of mobility, H2B remains bound to chromatin longer than our imaging time. As observed from the trajectory level RL analysis, H2B molecules that exhibit lower MSDs (RL group 1) tend to remain in the lowest-mobility state, while molecules that exhibit higher MSDs (RL group 2) switch between two mobility states. Accordingly, TF molecules that are bound to less mobile chromatin show limited switching and histone-like dwell times. However, TFs bound to more dynamic chromatin (with overall higher MSDs and more frequent transitions between the mobility states) show similar mobility transitions but are not as long-lived (exhibiting power-law dwell times). Thus, while state switching is reflective of the dynamics of the underlying chromatin mobility, the overall dwell times are reflective of the binding, which can be either long-lived (tightly bound) or power-law (sampling a broad range of affinities). Measuring TF dwell times in each mobility group under appropriate biological perturbations might help distinguish between different types of binding, which cannot be done from ensemble measurements of dwell times.

In this study, we have focused on long-binding events. A long-standing question in the field is how TFs scan the 4D genome in search of their binding sites. TF motifs are typically 8 to 20 bp in length and are embedded within a sea of nonspecific sequences (*5*). Theoretical considerations show that if TFs were to rely solely on Brownian motion to encounter their binding sites, they would take days to find a single specific binding site (*5*). Biophysical models of this apparent paradox suggest that bulk diffusion allows TFs to localize close to their specific sites, following which, they rely on 1D sliding, facilitated diffusion, and hopping to find their target motifs (*64*, *65*). While these models are very provocative, little direct experimental evidence is currently available. As imaging technologies develop and we push temporal and spatial resolutions to scales that are relevant for these processes, analysis tools presented here can help uncover modes of motion that remain elusive in conventional SMT studies. For example, using fast imaging, CTCF was shown to use anomalous diffusion along with transient trapping in CTCF clusters to boost its search efficiency, an interaction that depended on CTCF’s internal RNA binding region (*26*). Applying these techniques to study TF dynamics in the context of development, disease, and evolution can provide a window into fundamental biological processes through the lens of individual TFs, paving the way for the development of targeted therapeutics for diseases driven by TFs gone awry.

### Limitations

To get a complete picture of TF dynamics from search to binding, we must be able to image with very high spatiotemporal resolution. Sparse labeling allows us to achieve sub-pixel localization, but our temporal resolution still suffers from photobleaching. By fitting the H2B survival distribution to a triple exponential function as described previously (*17*), we estimate our photobleaching rate to be 13.03 ± 0.15 s (~9 sub-tracks), which is adequate for all the measurements reported in this study. We mitigate some of this by imaging with longer dark periods (200 ms) to capture long-lived binding events. However, this does not allow us to capture fast diffusing molecules because they move out of the imaging volume on these time scales. As described in Materials and Methods, the probability of a freely diffusing GR molecule to remain in the focal volume during our sampling interval is less than 10^−9^. Faster imaging will be required to quantitatively assess these states. Because of this, we restricted our analysis to the two lowest-mobility states. The mobility characteristics of slow-moving bound molecules should not depend on photobleaching and differential defocalization of the two low-mobility states. However, we note that TF molecules with different overall mobilities do exhibit distinct temporal dynamics (Fig. 6L). MINFLUX tracking (*66*, *67*) is currently the most promising nanoscopy technique that offers nanometer-scale spatial resolution with a temporal resolution of hundreds of microseconds. Developments in fluorophore chemistry that improve the brightness and photostability of fluorophores will make longer imaging more feasible on instruments such as MINFLUX, and researchers will be able to interrogate both long- and short-time behaviors in the same set of tracks.

While our analysis provides evidence for two distinct mobility states in the nucleus, our MSD curves span only six time lags. With only six time lags, we cannot comment on the nature of the mobility states. To use the MSD to reliably distinguish between different physical models such as sub-diffusion, fractional Brownian motion, and confined diffusion, we need at least three decades of time lags (*68*). Non-MSD approaches to estimate diffusive parameters perform better than traditional MSD analyses but still require at least two decades of time lags (*69*). It is possible to achieve these long time scales by tracking sub-nuclear structures like telomeres, which can be labeled by the binding of multiple fluorescent proteins such as telomeric repeat factor 2 (TRF2) (*70*). However, photobleaching keeps these time scales outside the purview of SMT experiments. As can be seen from our analysis of long tracks, even with 200-ms dark periods, we can only span a 20-fold range of time lags.

Our study and all the SMT studies cited here (*13*–*16*, *23*–*27*, *29*, *30*, *46*, *47*) have been conducted in 2D cross-sections of the nucleus, and it is possible for diffusing molecules to appear confined when projected in 2D. The higher-mobility states recovered from pEMv2 for most TFs (colored green and yellow in all the figures) could represent a combination of this population of diffusive molecules along with tracking errors. This is supported by the fact that the proportion of these states is unchanged under all the perturbations. The only way to conclusively determine what these states represent will be to perform fast 3D tracking. While we have shown here that 2D tracks of H2B and TFs do not exhibit any large-scale spatial separation, 3D tracking will be essential to test whether there could be any smaller-scale spatial separation of different mobility states.

Last, 2D tracking poses another notable challenge. When imaging molecules at the nuclear periphery or in perinucleolar regions, these molecules will undergo diffusion along an effective 2D surface. When these events are imaged in 2D, we are looking at the 1D intersection of the surface and the focal plane. These events will preferentially appear to be in a very low-mobility state because this is effectively 1D motion. One must be careful to attribute these to the more compact nature of heterochromatin (*15*) without performing appropriate comparisons with 3D tracking.

## MATERIALS AND METHODS

### Cell lines and cell culture

3617 mouse adenocarcinoma cells (*20*) were grown in high-glucose Dulbecco’s modified Eagle medium (DMEM; Gibco, #11960044) supplemented with 10% fetal bovine serum (FBS), 2 mM l-glutamine (Gibco, #25030081), 1% MEM non-essential amino acids (Gibco, #11140050), and 1 mM sodium pyruvate (Gibco, #11360070) at 37°C in a CO_2_ controlled incubator. 3617 cells contain stably integrated GFP-GR under a tetracycline-off system (*71*). To prevent the expression of GFP-GR, these cells were grown in the presence of tetracycline (5 μg/ml).

The 3T3-L1 mouse pre-adipocyte cell line (ATCC) was cultured in DMEM supplemented with 10% calf serum (Gibco, #26170043), 1% MEM non-essential amino acids, 1 mM sodium pyruvate, penicillin (50 U/ml), and streptomycin (50 μg/ml; Gibco, #15070063) at 37°C in a CO_2_ controlled incubator.

### Animal experiments

No animals were used in this work.

### Plasmid constructs

#### H2B

pHalo-H2B was generated by polymerase chain reaction (PCR) amplification of the H2B coding region from an H2B-GFP template and cloned into a pFC14A backbone (Promega, Madison, WI, USA) to fuse the HaloTag to the C terminus of H2B (*31*).

#### Steroid receptors

The pHaloTag-GR plasmid expresses rat GR fused to HaloTag (Promega, Madison, WI, USA) in the C terminus regulated by a CMVd1 promoter and has been described previously (*72*). pHalo-PR expresses human PR isoform beta fused with HaloTag at the N terminus, regulated by a CMV promoter (*24*). pHalo-PR open reading frame (ORF) clone was purchased from Promega (Promega, #FHC24423). pHalo-ER expresses human ERα fused to HaloTag in the C terminus regulated by a CMVd1 promoter and has been described previously (*24*, *73*). pHalo-AR expresses human AR with HaloTag fused to the C terminus. This plasmid was custom-made by Promega and has been reported previously (*24*).

#### PPARγ2 and mutants

pHalo-PPARγ2 expresses human PPARγ isoform 2 fused to HaloTag in the N terminus under a CMVd1 promoter (Promega ORF clone #FHC08305). PPARγ2 mutants were generated by nucleotide substitution using the QuikChange II XL Site Directed Mutagenesis Kit (StrataGene, La Jolla, CA, USA) following the manufacturer’s protocol. PCR primers were designed using QuikChange Primer Design Program. The primer sets used to generate the mutants were as follows: PPARγ2-C159S (DBDmut) (1, 5′-CCGGAAGAAACCCTTGGATCCTTCACAAGCATG-3′ and 2, 5′-CATGCTTGTGAAGGATCCAAGGGTTTCTTCCG-3′) and PPARγ2-L470R (HETmut) (1, 5′-CCGTGACAATCTGTCTGCGGTCTGTCATTTTCTGG-3′ and 2, 5′-CCAGAAAATGACAGACCGCAGACAGATTGTCACGG-3′). All mutations were verified by sequencing.

#### Co-regulators

pHalo-RELA expresses human NF-κB subunit p65 fused with HaloTag at the N terminus in a pFN22K backbone. This construct was purchased from Promega. pHalo-GRIP1 expresses mouse GRIP1 with an N terminus HaloTag fusion regulated by a CMVd1 promoter. This was generated by PCR amplification of the GRIP1 coding region from an EGFP-GRIP1 template and subsequent cloning into a pFN22K backbone using Sgf I and Pme I restriction sites (*24*). pHalo-SMARCA4 expresses human SMARCA4 with HaloTag fused to the N terminus under a CMVd1 promoter (Promega ORF FHC12075). pHalo-MED26 expresses human MED26 fused with a HaloTag at the N terminus and was a gift from J. Conaway’s laboratory. pHalo-CTCF expresses mouse CTCF with HaloTag fused to the C terminus. This was generated by PCR amplification of the CTCF coding region from a CTCF-EGFP template (*74*) and cloned into the pHalo-GR backbone, which was cut using the Pvu I and Xho I restriction enzymes (New England Biolabs, Ipswich, MA) and has been described previously (*17*).

#### EGFP construct

EGFP-C/EBPα expresses rat C/EBPα with an EGFP fusion on the N terminus (this was a gift from F. Schaufele, University of California San Francisco, San Francisco, CA, USA) and has been described previously (*75*).

### Transient transfections and agonist treatments

3617 and 3T3-L1 cells were plated in LabTek II (Thermo Fisher Scientific, Waltham, MA, USA) or Cellvis (Mountain View, CA, USA) chamber slides for 24 hours before transfection. For 3617 cells, the indicated plasmids were transiently transfected using jetPRIME reagent (PolyPlus, New York, NY, USA) following the manufacturer’s protocol. The protocol was optimized to prevent overexpression of HaloTag-protein chimeras (*24*). Cells were incubated in the jetPRIME reaction mixture containing 500 ng of DNA for 4 hours. The medium was then replaced with phenol red–free DMEM containing charcoal-stripped FBS (Life Technologies, Carlsbad, CA, USA) supplemented with 2 mM l-glutamine, 1% MEM non-essential amino acids, 1 mM sodium pyruvate, and tetracycline (5 μg/ml), and the cells were allowed to recover overnight.

For 3T3-L1 cells, 24 hours after plating, the medium was changed to optiMEM (Gibco, #31985070), and the cells were transfected with the indicated HaloTag- and/or EGFP-protein chimeras using Lipofectamine 2000 reagent (Invitrogen, Waltham, MA, USA) following the manufacturer’s protocol. Briefly, for HaloTag-protein fusions, we used 750 ng of DNA per 100 μl of Lipofectamine 2000 transfection mix. For EGFP-protein constructs, we used 4.5 μg of DNA per 100 μl of transfection mix. After incubating the cells in the transfection mix for 4 hours, the medium was replaced with fresh phenol red–free growth medium, and the cells were allowed to recover overnight.

Before imaging, the cells were incubated in a medium containing 5 nM Janelia Fluor 549 (JF_549_) HaloTag ligand (*21*, *59*) for 20 min. The cells were then washed three times with phenol red–free medium and returned to the incubator for 10 more minutes. Cells were then washed once more. 3617 cells were either left untreated or treated with the indicated hormone (100 nM): dexamethasone (Dex), 17β-estradiol (E_2_), dihydrotestosterone (DHT), or progesterone (Prog) for 20 min before imaging. Dex, E_2_, DHT, and Prog were purchased from Sigma-Aldrich (St. Louis, MO, USA). 3617 cells expressing Halo-RELA were treated with tumor necrosis factor–α (TNFα; 30 ng/ml; Sigma-Aldrich, St. Louis, MO, USA) for 30 min before imaging. 3T3-L1 cells were all treated with 1 μM BRL49653/rosiglitazone (Rosi; Cayman Chemical Company, Ann Arbor, MI, USA) for 1 hour. Between 2 and 10 biological replicates were collected per condition.

### Microscopy

All samples were imaged on a custom-built HILO microscope in the LRBGE Optical Microscopy Core at the National Cancer Institute (NCI), National Institutes of Health (NIH). Detailed information can be found in (*59*). Briefly, the microscope has a 150× 1.45 numerical aperture objective (Olympus Scientific Solutions, Waltham, MA, USA); an Okolab stage-top incubator for temperature, and 5% CO_2_ control (Okolab, Pozzuoli NA, Italy). The microscope is equipped with a 561-nm laser (iFLEX-Mustang, Excelitas Technologies Corp., Waltham, MA, USA) and an acousto-optical tunable filter (AOTFnC-400.650, AA Optoelectronic, Orsay, France) (*22*, *59*). Images were collected using an EM-CCD camera (Evolve 512, Photometrics, Tucson, AZ, USA) every 200 ms (5-Hz frame rate) with an exposure time of 10 ms for a total of 2 min (600 frames) with a laser power of 0.96 mW (*17*). The pixel size for this setup is 104 nm.

### Tracking

Particle detection and tracking are performed using TrackRecord v6, a custom tracking software written in MATLAB (version 2016a; The MathWorks Inc., Natick, MA, USA) that is publicly available at Zenodo (https://doi.org/10.5281/zenodo.7558712) and has been extensively described previously (*17*, *23*, *24*, *31*, *59*). The image stacks were filtered using Top-hat, Wiener, and Gaussian filters. A hand-drawn region of interest was used to demarcate the boundary of the nucleus. The particle detection intensity threshold was determined to be the lowest threshold at which less than 5% of detected molecules had a signal-to-noise ratio of 1.5 or less. Sub-pixel localization was achieved by fitting the detected particles to a 2D Gaussian. Detected particles were then tracked using a nearest-neighbor algorithm (*76*) with a maximum allowed jump of 4 pixels (416 nm), a maximum allowed gap of one frame, and a shortest track of six frames. A maximum jump of 4 pixels (416 nm) has been validated previously for bound molecules (*31*).

For all the tested proteins, we localize 10 to 40 molecules at the beginning of the movie, with decreasing localizations at later time points, due to photobleaching (fig. S1). Out of these detected molecules, the number of molecules that are tracked depends on the bound fraction of the molecule. For molecules with a high bound fraction (e.g., H2B in both cell lines), most of the detected molecules can be tracked (fig. S1, A and B). On the other hand, for molecules with small bound fractions (e.g., untreated SRs and PPARγ2 mutants), only a small fraction of detected molecules are tracked (fig. S1, C, E, G, I, and Q to S).

To confirm that tracking errors were minimal under our experimental conditions, we performed the following quality control steps. First, we calculated the distance of every tracked particle to the two nearest-neighbor particles in the next frame. Table S1 shows the percentage of tracked molecules for which the second nearest neighbor falls within 416 nm (the allowed maximum jump) of the tracked molecule. As can be seen from table S1, for all the tested molecules, the average fraction of second nearest neighbors that fall within 416 nm of a tracked molecule is 0.73%, with the highest fraction being 1.54% for the AR activated by DHT. These data show that the frequency of tracking errors due to labeling density and the tracking parameters is <1% in most cases and no higher than 1.54%.

Next, we compared the effect of allowing a gap of one frame (the default parameter used for all the data presented here), with the more stringent gapless tracking (gap = 0). We tracked histone H2B and the GR with both gap = 1 and gap = 0 and compared the survival distributions and ensemble MSD. We find that regardless of the gap parameter, the recovered survival distribution and MSD are identical (fig. S2). Furthermore, the MSD for H2B shows good agreement with previously published data (*14*, *31*).

Since we are imaging at a relatively slow frame rate of 200 ms, it is theoretically possible that we are linking different molecules between consecutive frames. To confirm that this is not the case, we next checked for occurrences of the following type of error: If we localize molecule A in frame *n*, what is the frequency of molecule B appearing in the 200-ms interval between frame *n* and *n + 1* and molecule A leaving the focal volume in the same period? This will result in a track segment linking molecule A in frame *n* to molecule B. To measure the frequency of such errors, we imaged both H2B and GR at fast frame rates (12 ms). This allows us to observe the arrival of new molecules within 416 nm of the original localization for multiple frames up to 200 ms. A potential source for misconnection errors would be the presence of multiple particles within 416 nm of the original localization in the intervening frames with only one molecule at the remaining 200 ms.

For every 200 ms segment that had one molecule at *t* = 0 and exactly one molecule at *t* = 200 ms within 416 nm of the original localization, we counted the number of times more than one particle is detected within 416 nm of the original localization during this period.

We estimated an upper bound for this type of error as

$$E=\frac{\#\;\text{segments with}>1\;\text{particle within}\;416\;\text{nm of original localization}}{\mathrm{Total}\;\#200\;\text{ms segments with}\;1\;\text{localization at the beginning and end}}$$

We note that this is an upper bound because if we have more than one particle within 416 nm of the original localization, either the single particle at 200 ms could be the original particle or it could have been replaced by another particle and we cannot distinguish between these possibilities.

For H2B, we find E_H2B_ = 2.044% and for GR, E_GR−Dex_ = 2.219%. This is an estimate for tracking errors over a 200-ms time scale. For these errors to compound across our mobility analyses, these errors must occur for six consecutive 200-ms segments, for which this error probability would be multiplicative (assuming that blinking or the arrival of molecule B and disappearance of molecule A is independent for each 200-ms interval, which is quite reasonable) and hence infinitesimally small. These data show that our tracking parameter choices result in minimal tracking errors for data acquired every 200 ms.

### Defocalization probability

The probability of a molecule with diffusion coefficient *D* remaining within the detection range Δ*z* after time Δ*t* can be explicitly calculated as (*25*)

$$P_{\mathrm{remaining}}(D,\text{Δ}t)=\frac{1}{\text{Δ}z}\int_{-\text{Δ}z/2}^{\text{Δ}z/2}\left\{1-\sum_{n=0}^{\infty }{\left(-1\right)}^{n}\left[erfc\left(\frac{\frac{(2n+1)\text{Δ}z}{2}-z}{\sqrt{4D\text{Δ}t}}\right)+erfc\left(\frac{\frac{(2n+1)\text{Δ}z}{2}+z}{\sqrt{4D\text{Δ}t}}\right)\right]\right\}dz$$

We estimated our depth of focus to be ~400 nm on the basis of the procedure described in (*77*). Freely diffusing GR has a diffusion coefficient of 1.8 μm^2^/s (*59*). Using the above equation, the probability of freely diffusing GR remaining within our 400 nm detection range at 200 ms is less than 10^−9^.

### Localization precision

For a particle that undergoes normal diffusion with a diffusion coefficient *D_k_*, the diagonal covariance matrix element Σ*_k_* is given by (*28*, *78*, *79*)

$${\text{Σ}}_{k}=2D_{k}\text{Δ}t+2\sigma_{k}^{2}-4RD_{k}\text{Δ}t$$

where $R\approx \frac{1}{6}\cdot \frac{\text{Δ}t_{\mathrm{exposure}}}{\text{Δ}t}$ is the motion blur coefficient.

pEMv2 outputs the covariance matrix Σ*_k_*(*i*, *i*) and the optimal diffusion coefficients *D_k_* for each state *k*. We can rearrange the above equation to calculate the localization error as

$$\sigma_{k}=\sqrt{\frac{1}{2}\;\cdot [{\text{Σ}}_{k}-2D_{k}\text{Δ}t(1-2R)]}$$

Including motion blur, pEM estimates the localization precision to be ~20 nm for state 1 and ~40 nm for state 2. The higher-mobility states have a localization precision of ~70 nm.

### Identification of distinct diffusive states using pEMv2

pEMv2 (*28*) was used to classify the single-molecule trajectories into multiple diffusive states. pEMv2 requires tracks to be divided into sub-tracks of equal length. We split our trajectories into sub-tracks of length of seven frames because longer tracks increase the likelihood of transitions within a sub-track. We ran pEMv2 independently on each protein and treatment to avoid forcing different datasets to converge on the same mobility states. No prior assumptions on the number of diffusive states or the types of diffusive motion were made (*28*). pEMv2 was allowed to explore between 1 and 15 states, with 20 reinitializations and 200 perturbations. The maximum number of iterations was set to 10,000 with a convergence criterion of 10^−7^ for the change in the log-likelihood function. The convergence of pEMv2 was verified through multiple runs. The covariance matrix was allowed to have three features.

After the classification by pEMv2, each sub-track is assigned a posterior probability to belong to each of the states. For example, if pEMv2 converges to three states, then each sub-track would have three posterior probabilities, one for each determined state. We assign each sub-track to the state for which it has the highest posterior probability (fig. S4A).

A sub-track could have similar posterior probabilities to belong to two or more states. For instance, in our mock example with three states (fig. S4A), we could have a sub-track with a posterior probability distribution of (0.9, 0.05, 0.05), in which case, we would assign the sub-track to state 1. However, we could also have a sub-track with a posterior probability distribution of (0.5, 0.4, 0.1); in which case, while we would assign the sub-track to state 1, it has a very high probability to belong to state 2 as well. To mitigate this, we calculated ΔPP, which is the difference between the two highest posterior probabilities for each sub-track, and excluded sub-tracks with ΔPP ≤ 0.2 from the ensemble MSD and population fraction calculations (fig. S4B).

### Calculation of the unbound fraction

States that account for less than 5% of all sub-tracks are excluded from the calculation of the population fraction (fig. S4C). For consistent comparison of population fractions of steroid receptors before and after hormone treatment or PPARγ2 wild type against mutants, we needed an estimate of the unbound fraction. Following the methodology outlined in (*17*, *31*), we used the respective H2B jump histograms to calculate two jump distance thresholds for each cell line: *R*_min_ is the jump distance of 99% of H2B molecules between consecutive frames, and *R*_max_ is the jump distance of 99% of H2B molecules between six frames (equal to the shortest track). Jump events larger than *R*_min_ over consecutive frames or larger than *R*_max_ over six frames were classified as unbound. For each species, the unbound fraction was then calculated as the ratio of the total number of unbound events to the total number of tracked molecules. For 3617 cells, *R*_min_ = 250 nm and *R*_max_ = 330 nm. For 3T3-L1 cells, *R*_min_ = 270 nm and *R*_max_ = 390 nm.

### Photobleaching corrected survival distributions

The photobleaching rate was calculated using a previously published methodology (*17*). Briefly, the raw H2B survival distribution was fit to a triple exponential function, with the slowest exponential parameter being the photobleaching rate.

The raw survival distributions for the two RL groups were calculated as follows. If *S*(*t*) is the raw survival distribution and *k*_PB_ is the photobleaching rate, then the photobleaching-corrected survival distribution $\hat{S}(t)$ is given by

$$\hat{S}(t)=\frac{S(t)}{e^{-k_{\mathrm{PB}}t}}$$

### Transition probabilities

For the calculation of transition probabilities, because most of the tracks belong to low-mobility states 1 and 2, all the other states detected by pEMv2 were grouped together into a third “other” state. This allows us to calculate the transition probability among three states: low-mobility state 1, low-mobility state 2, and “other” states. For each track, the number of transitions between each pair of these states is calculated using a custom MATLAB script. Only tracks with at least three sub-tracks were included in this analysis. A total of 26 ± 5% of tracks have at least three sub-tracks, but these account for 67 ± 6% of total sub-tracks (table S2). These transition counts are then added up to obtain a transition matrix *T* where the element *T*(*i*, *j*) is the number of transitions from state *i* to state *j*. This matrix is then normalized to obtain the transition matrix *P_t_*, where

$$P_{t}(i,j)=\frac{T(i,j)}{\sum_{j=1}^{3}T(i,j)}$$

To test whether these transition probabilities are different from those recovered from a randomized ensemble with the same population fraction, the sub-track state assignments are randomly shuffled, and the transition probabilities ${\hat{P}}_{t}(i,j)$ are calculated for this randomized ensemble. This process is repeated 1000 times, and the statistical significance for a transition probability *P_t_*(*i*, *j*) is reported as the proportion of randomized trials with ${\hat{P}}_{t}(i,j)>P_{t}(i,j)$.

### Estimation of diffusion coefficients and localization errors using Spot-On

Spot-On (*25*) was used to fit a two-state model to the cumulative distribution function of displacements of pEMv2 classified sub-tracks. The following parameters were used: binWidth = 0.01, UseWeights = 1, MaxJump = 5.05, FitIterations = 3, JumpsToConsider = 6, and TimePoints = 4. On the basis of the pEMv2 results, the range for the diffusion coefficient fit was set to [0.0001, 0.05].

### Estimation of the MSD distribution using the RL algorithm

The single particle tracking data were used to calculate the self-part of the vHc as *G_s_*(*r*, τ) = *A_s_*〈δ(***r_i_*** − ∣***r_i_***(*t* + τ) − ***r_i_***(*t*)∣〉, where ***r***_***i***_ is the position of the *i*-th nucleosome and *A_s_* = ∫ *d*^2^*rG_s_*(*r*, τ) is a normalization constant. The vHc is assumed to be a superposition of Gaussian functions, $q(r,M)=\left(\frac{1}{\pi M}\right)\exp\left(-\frac{r^{2}}{M}\right)$ as *G_s_*(*r*, τ) = ∫ *P*(*M*, τ)*q*(*r*, *M*)*dM*, where *P*(*M*) is the distribution of MSDs of the population of nucleosomes. The RL algorithm is used to extract *P*(*M*) from the empirical vHc as follows (*16*): from an initial distribution, $P^{0}(M)=\exp\left(-\frac{M}{M_{0}}\right)$, *P*^*n*+1^(*M*, τ) at the (*n* + 1)-th iteration was iteratively obtained from

$$P^{n+1}=P^{n}\int \frac{G_{s}(r,\tau )}{{G^{n}}_{s}(r,\tau )}q(r,M)d^{2}r$$

with the constraint that *P^n^*(*M*, τ) > 0 and normalized. The minima of *P*(*M*, τ) were used to classify individual nucleosome tracks into different mobility states.

## Notes

View/request a protocol for this paper from *Bio-protocol*.
