# Supplementary material for: Dynamic switching of transcriptional regulators between two distinct low-mobility chromatin states
Source: Sci Adv. 2023 Jun 14;9(24):eade1122. doi: 10.1126/sciadv.ade1122 (PMC10954219; doi:10.1126/sciadv.ade1122)
Supplement: Supplementary file 1 — Figs. S1 to S11 Tables S1 and S2 Legends for movies S1 to S4 [file sciadv.ade1122_sm.pdf]

Science Advances
AAAS

Supplementary Materials for

Dynamic switching of transcriptional regulators between two distinct low-mobility chromatin states

Kaustubh Wagh *et al.*

Corresponding author: Diego M Presman, presmandm@fbmc.fcen.uba.ar; Arpita Upadhyaya, arpitau@umd.edu; Gordon L Hager, hagerg@exchange.nih.gov

Sci. Adv. **9**, eade1122 (2023)
DOI: 10.1126/sciadv.ade1122

The PDF file includes:

Figs. S1 to S11
Tables S1 and S2
Legends for movies S1 to S4

Other Supplementary Material for this manuscript includes the following:

Movies S1 to S4

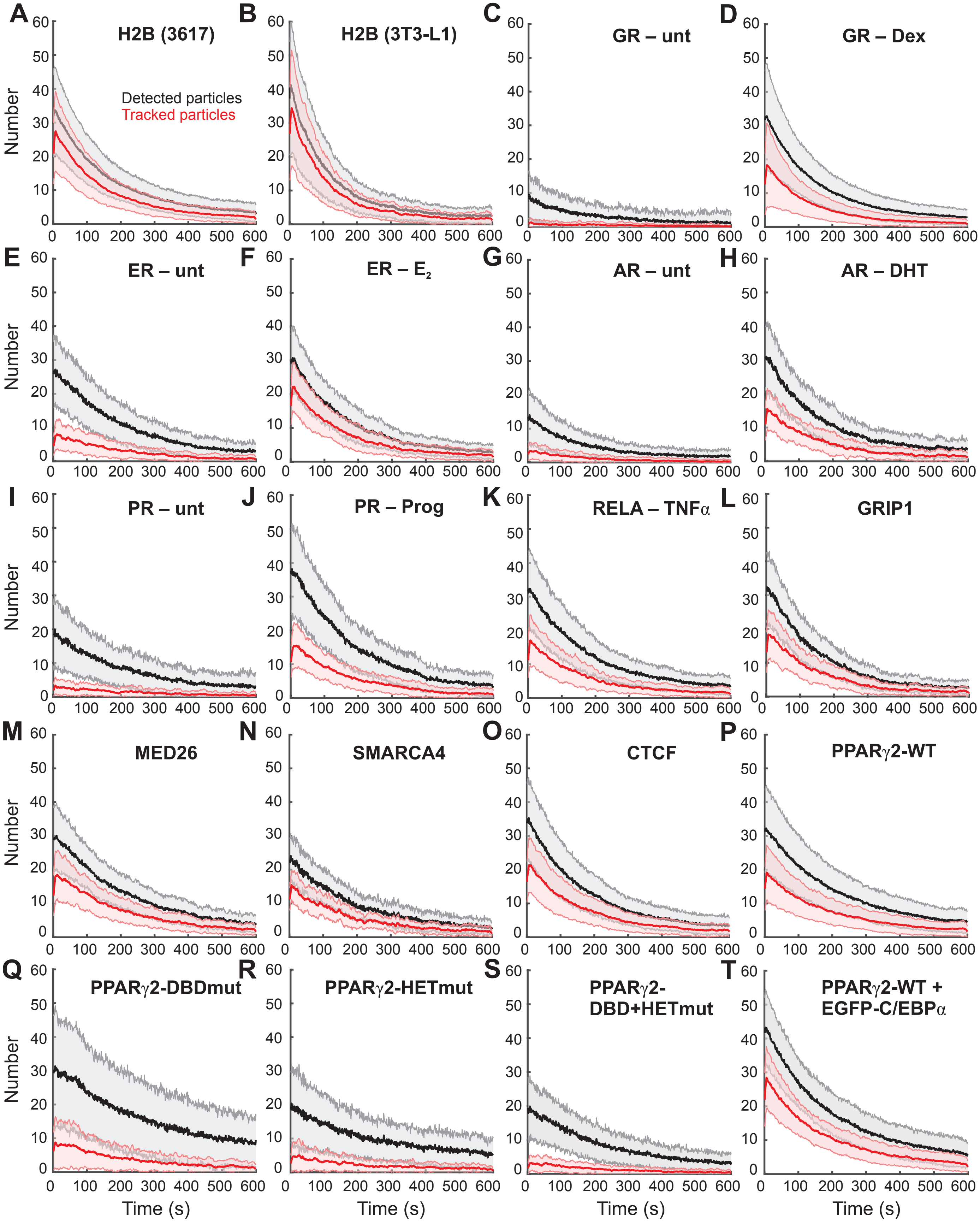

A
H2B (3617)
Detected particles
Tracked particles
B
H2B (3T3-L1)
C
GR – unt
D
GR – Dex
E
ER – unt
F
ER – E2
G
AR – unt
H
AR – DHT
I
PR – unt
J
PR – Prog
K
RELA – TNFα
L
GRIP1
M
MED26
N
SMARCA4
O
CTCF
P
PPARγ2-WT
Q
PPARγ2-DBDmut
R
PPARγ2-HETmut
S
PPARγ2-DBD+HETmut
T
PPARγ2-WT + EGFP-C/EBPα
Number
Time (s)

Figure S1

Fig. S1: Localization and track density per frame. Localization (black) and track (red) density per frame for the indicated molecules in the 3617 (panels **A, C – O**) and 3T3-L1 cell lines (panels **B, P – T**)**.** The shaded error bars indicate the standard deviation.

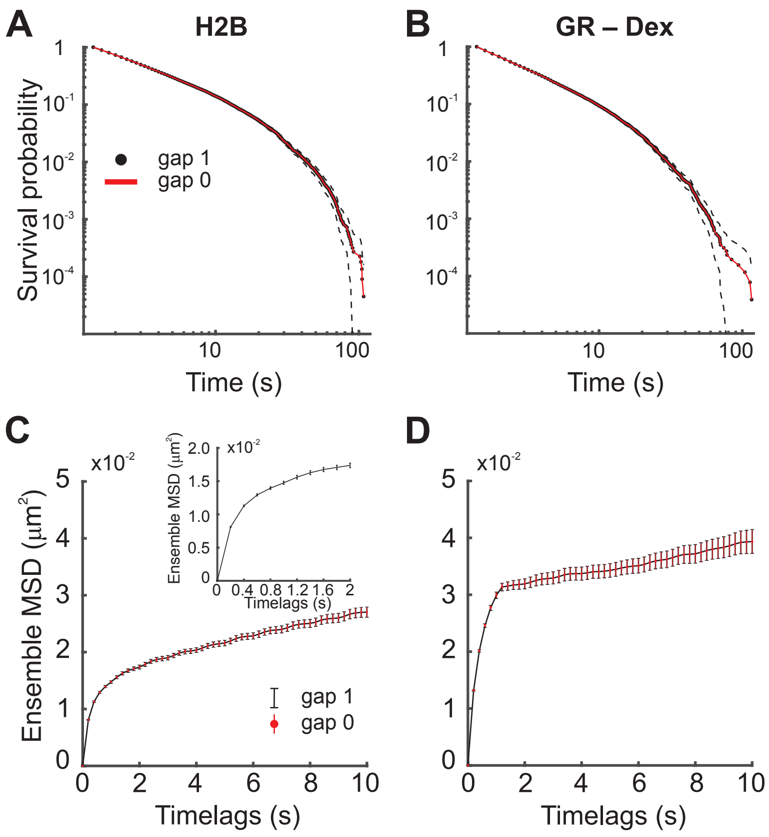
A
H2B
B
GR – Dex
Survival probability
gap 1
gap 0
Time (s)
C
D
Ensemble MSD (μm²)
x10⁻²
Timelags (s)
gap 1
gap 0

Figure S2

Fig. S2. Effect of changing the gap parameter. (A – B) Raw survival distributions of dwell times for **(A)** H2B and **(B)** the glucocorticoid receptor (GR) activated with dexamethasone (Dex) in 3617 cells. Black circles represent the survival distribution for data analyzed with gap = 1 (the standard in the manuscript) while the solid red lines represent that for data analyzed with gap = 0. The black dashed lines are the 99% confidence intervals for the survival distribution. **(C – D)** Ensemble mean squared displacement for **(C)** H2B and **(D)** GR activated with Dex. Black lines represent data tracked with gap = 1 and red lines represent data tracked with gap = 0. The error bars indicate the standard error of the mean. Inset in **(C)** shows a zoomed-in MSD plot for H2B tracked with gap = 1, for timelags of 0-2 s.

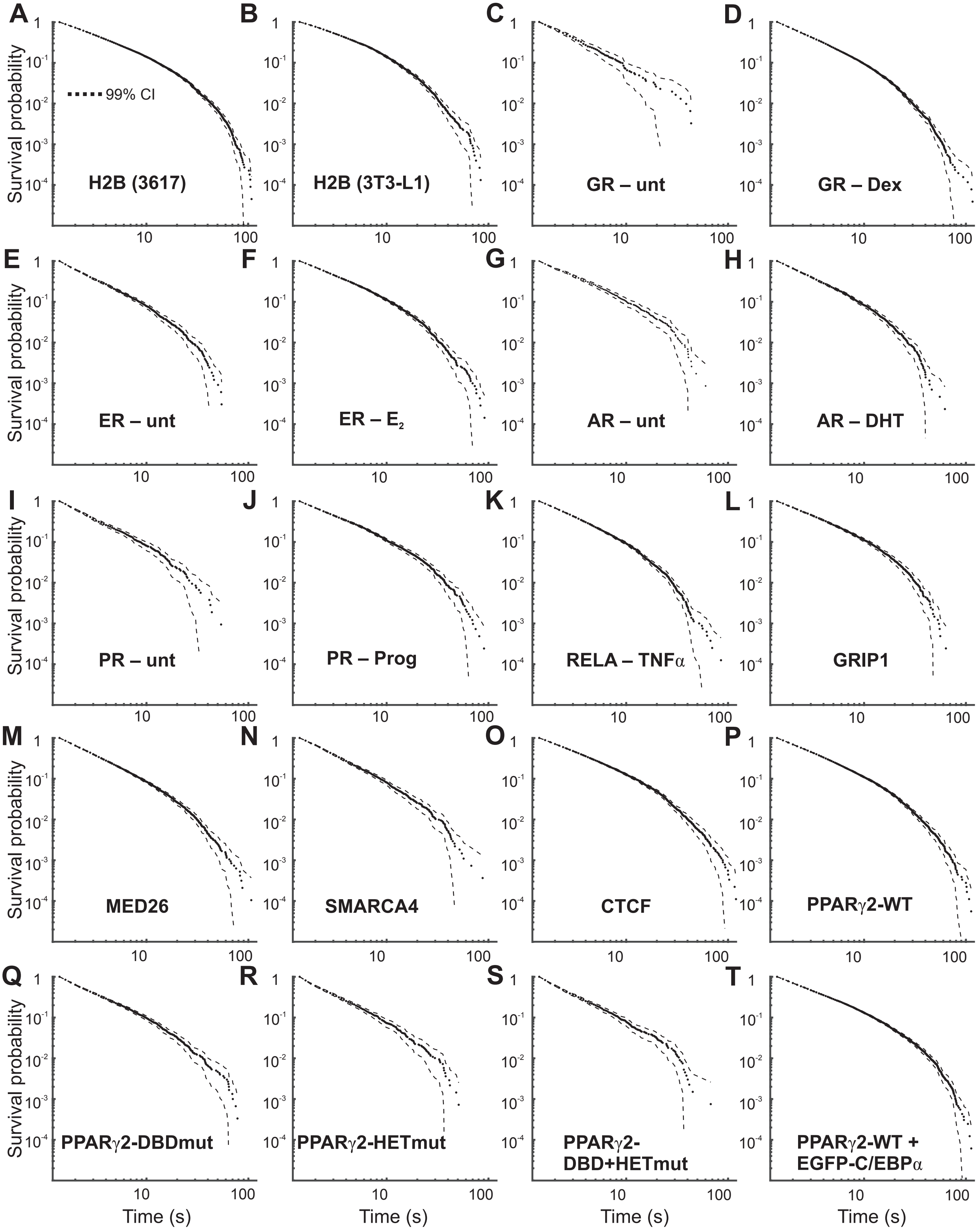
A
B
C
D
E
F
G
H
I
J
K
L
M
N
O
P
Q
R
S
T
Survival probability
Time (s)
99% CI
H2B (3617)
H2B (3T3-L1)
GR – unt
GR – Dex
ER – unt
ER – E_2
AR – unt
AR – DHT
PR – unt
PR – Prog
RELA – TNFα
GRIP1
MED26
SMARCA4
CTCF
PPARγ2-WT
PPARγ2-DBDmut
PPARγ2-HETmut
PPARγ2-DBD+HETmut
PPARγ2-WT + EGFP-C/EBPα

Figure S3

Fig. S3: Survival distribution of track lengths. Raw survival distribution of track lengths for indicated species in 3617 (panels **A, C – O**) and 3T3-L1 cell lines (panels **B, P – T**). The dashed lines represent the 99% confidence interval.

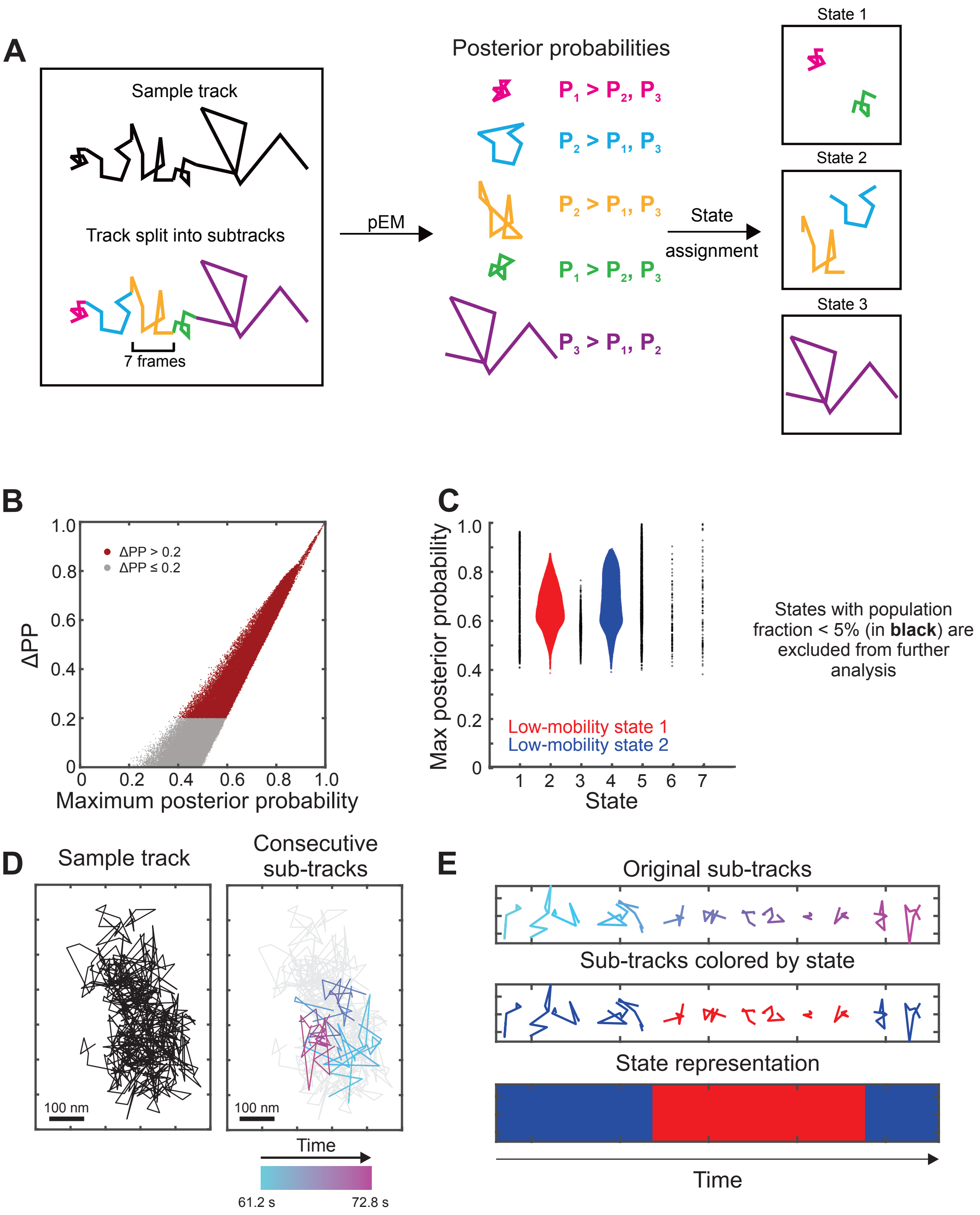
A
Sample track
Track split into subtracks
7 frames
pEM
Posterior probabilities
P1 > P2, P3
P2 > P1, P3
P2 > P1, P3
P1 > P2, P3
P3 > P1, P2
State
assignment
State 1
State 2
State 3
B
ΔPP > 0.2
ΔPP ≤ 0.2
ΔPP
Maximum posterior probability
C
Max posterior probability
Low-mobility state 1
Low-mobility state 2
State
States with population fraction < 5% (in black) are excluded from further analysis
D
Sample track
Consecutive sub-tracks
100 nm
100 nm
Time
61.2 s
72.8 s
E
Original sub-tracks
Sub-tracks colored by state
State representation
Time

Figure S4

Fig. S4: pEM workflow and data representation. (A) Cartoon showing pEM analysis workflow. Tracks are divided into sub-tracks of length 7 frames and classified into a state based on the posterior probability distribution. **(B)** Scatter plot of the difference of two highest posterior probabilities versus the maximum posterior probability. **(C)** Swarmchart of maximum posterior probability vs state (ordered in increasing order of diffusivity). States with population fraction less than 5% are colored in black and excluded from further analysis. **(D)** (left) Sample single molecule trajectory of the glucocorticoid receptor (GR) (the same track as shown in Fig. 3A, B). (right) Sub-tracks from 61.2s to 72.8s color-coded for time. **(E)** (top) Sub-tracks in panel D (right) separated in space for visualization (middle) Same tracks as in the top panel colored according to state assignment by pEMv2 (bottom) Block state representation to visualize state transitions. Each sub-track is represented by a red (state 1) or blue (state 2) pixel.

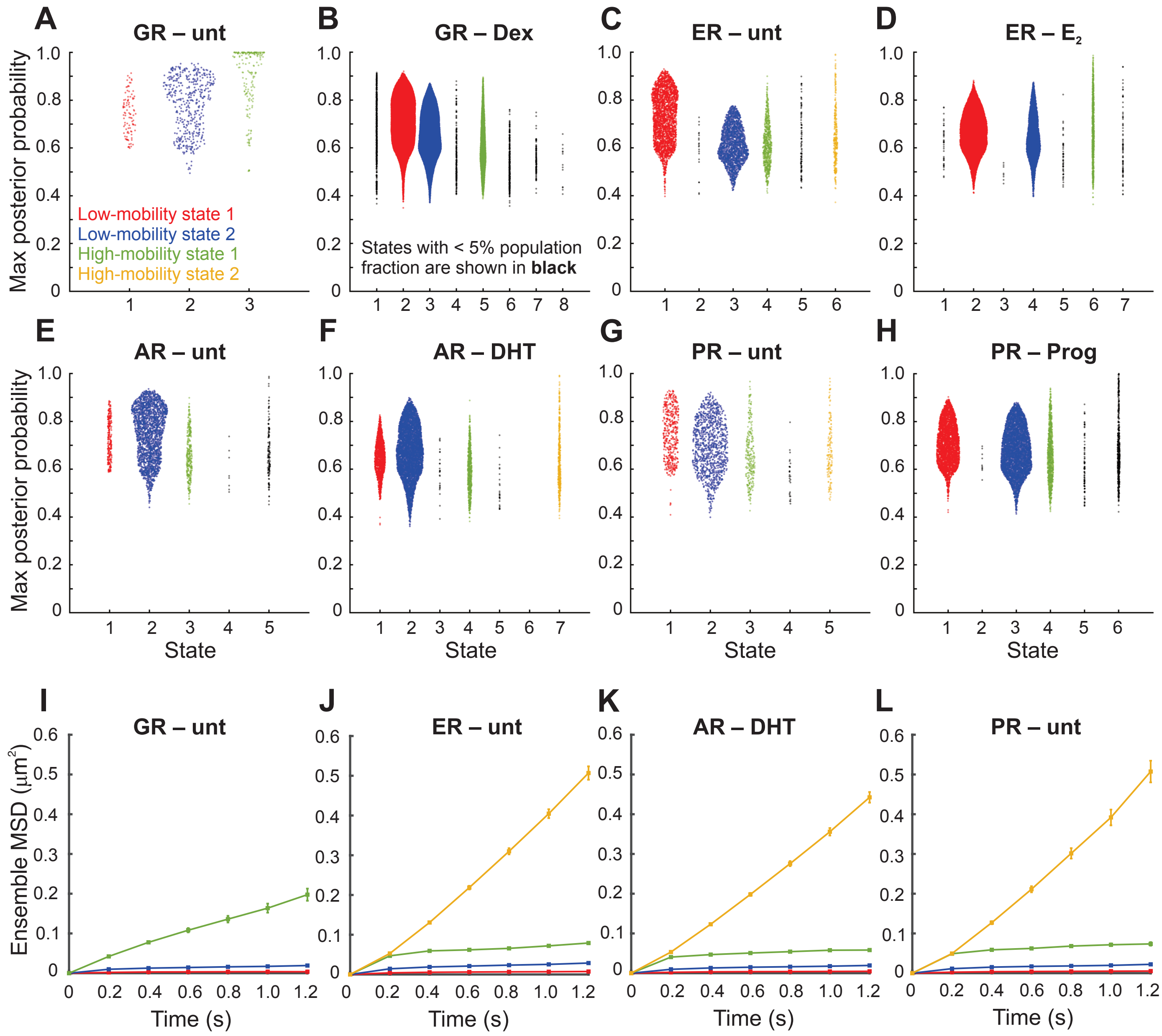
A
GR – unt
B
GR – Dex
C
ER – unt
D
ER – E2
Max posterior probability
1.0
0.8
0.6
0.4
0.2
0
Low-mobility state 1
Low-mobility state 2
High-mobility state 1
High-mobility state 2
States with < 5% population fraction are shown in **black**
E
AR – unt
F
AR – DHT
G
PR – unt
H
PR – Prog
State
I
GR – unt
J
ER – unt
K
AR – DHT
L
PR – unt
Ensemble MSD (μm2)
Time (s)

Figure S5

Fig. S5: Swarmcharts of steroid receptors and select mean-squared displacements. Maximum posterior probability vs pEMv2 state for **(A)** Glucocorticoid receptor (GR) without hormone, **(B)** GR activated with dexamethasone, **(C)** Estrogen receptor (ER) without hormone, **(D)** ER activated with 17β-estradiol (E_2), **(E)** Androgen receptor (AR) without hormone, **(F)** AR activated with dihydrotestosterone (DHT), **(G)** Progesterone receptor (PR) without hormone, **(H)** PR activated with progesterone (Prog). **(I – L)** Ensemble mean-squared displacement (MSD) plots for the indicated species showing the complete y-axis range for the higher mobility states.

Timelag = 0.8 s

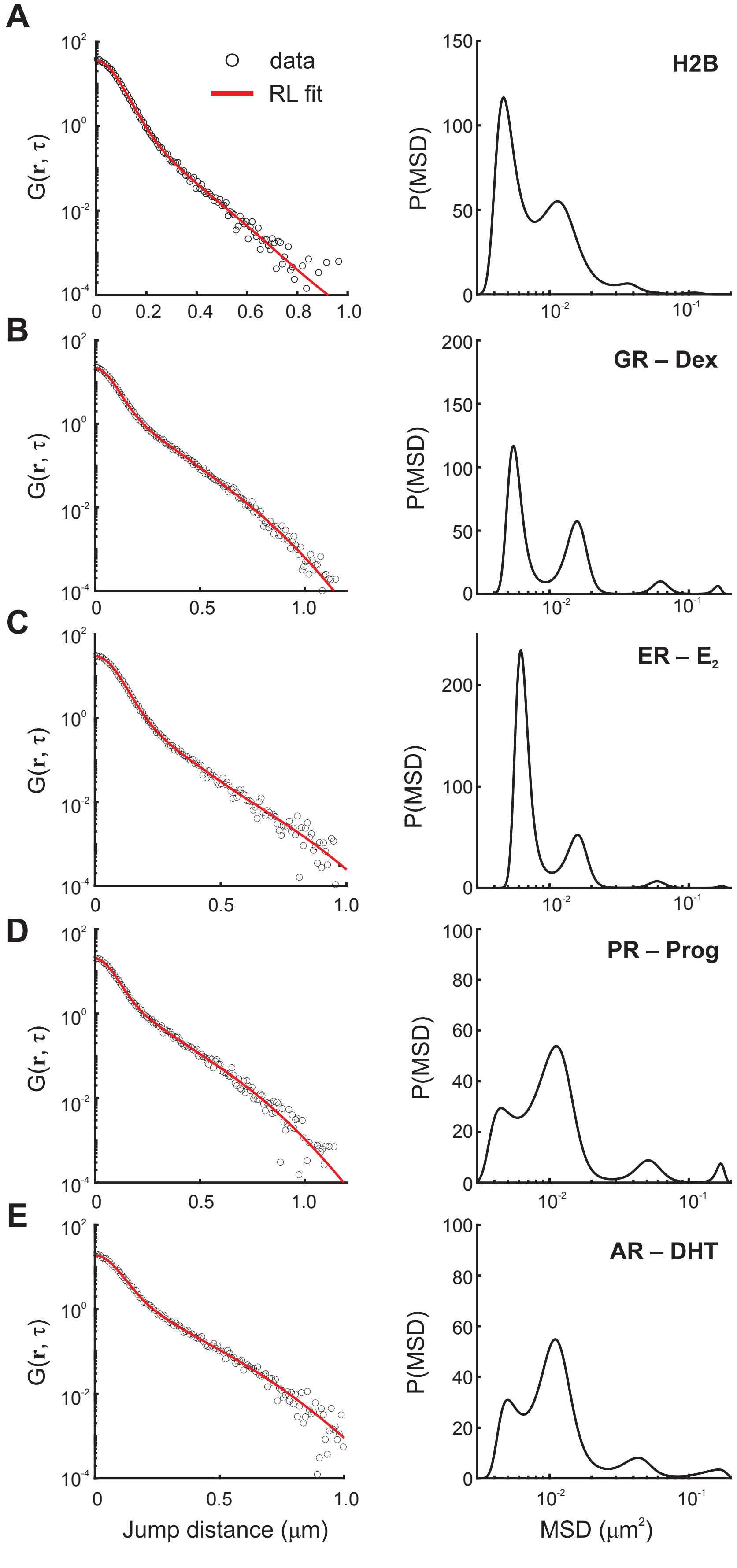

Figure S6 – Page 1

Timelag = 0.6 s

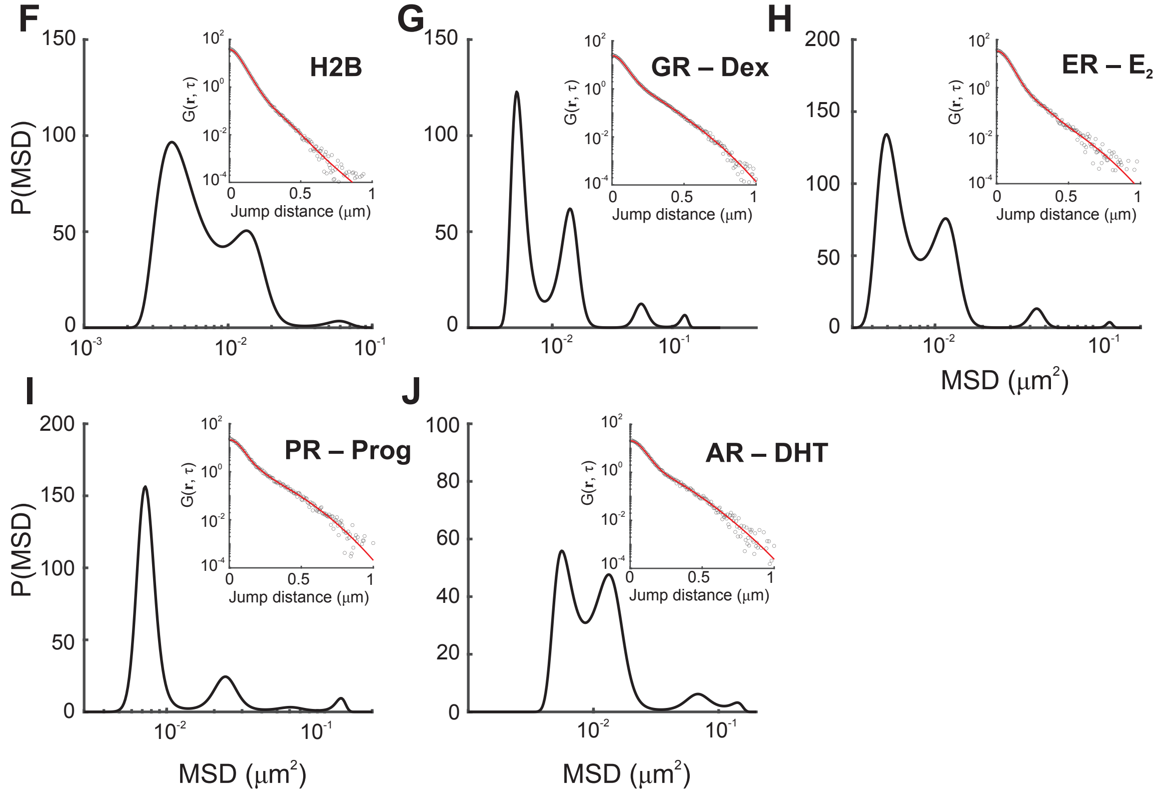

Timelag = 1 s

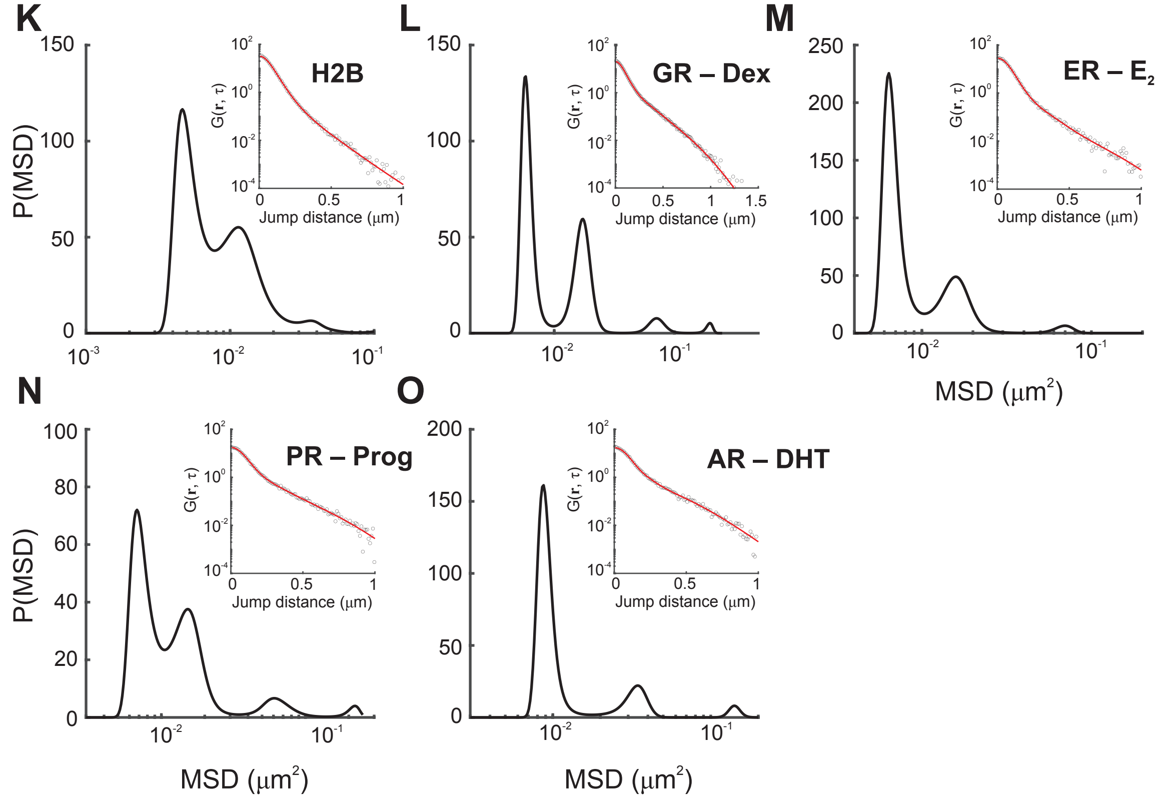

Figure S6 – Page 2

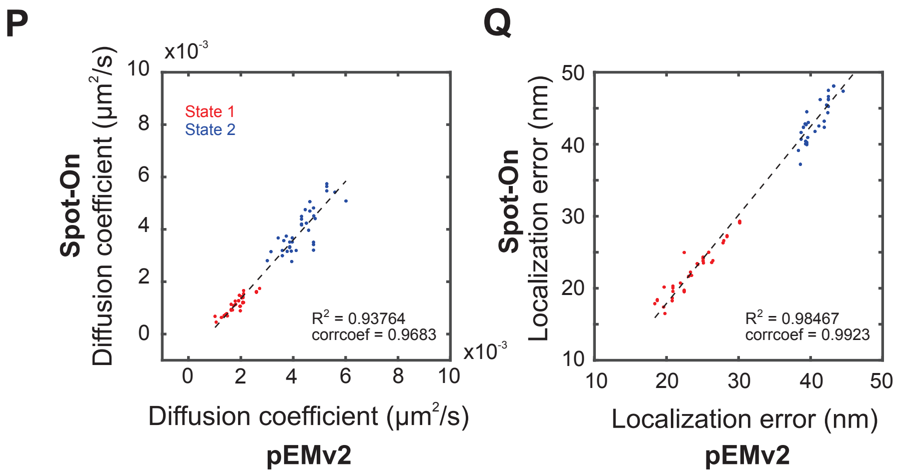

Figure S6 – Page 3

Fig. S6: Prediction of diffusivity distributions from van Hove correlations reveals distinct mobility states of transcription factors and histones. **(A – E) (left)** van Hove correlation (vHc) function G(r,t) for single molecule trajectories of **(A)** H2B, **(B)** GR activated with dexamethasone (Dex), **(C)** ER activated with 17β-estradiol (E_2), **(D)** PR activated with progesterone (Prog), and **(E)** AR activated with dihydrotestosterone (DHT), calculated at t=0.8 s. Red line is the computed vHc from the mean-squared displacement (MSD) histogram (P(MSD)) using Richardson-Lucy inversion. **(right)** The distribution P(MSD) computed at 0.8 s for the molecular species indicated. **(F – O)** P(MSD) computed at timelag of 0.6 s **(F – J)** and 1 s **(K – O)**; (insets) vHc for the indicated species. Red lines indicate the computed vHc from P(MSD) using Richardson-Lucy inversion. **(P)** Results from separately fitting displacement histograms of sub-tracks classified into each of state 1 and state 2 by pEMv2 to a two-state model using Spot-On. Scatter plot of estimated diffusion coefficients for the lowest two states for each tested dataset. Each circle represents the results of one cell line + protein + treatment condition. The x-axis represents diffusion coefficients recovered from pEMv2 and the y-axis represents those obtained by fitting a two-state diffusion model using Spot-On. The dashed line is a linear fit and the corresponding R^2 and correlation coefficient are indicated below the figure. Red circles indicate the lowest mobility state (state 1), and blue circles indicate the next lowest mobility state (state 2). **(Q)** Scatter plot of localization error estimated for sub-tracks belonging to state 1 (red) and state 2 (blue). The x-axis represents localization error calculated from the pEMv2 covariance matrix and the y-axis represents localization error calculated from Spot-On. Like in panel **P**, sub-tracks belonging to each pEMv2 state were analyzed separately and fit to a two-state diffusion model using Spot-On.

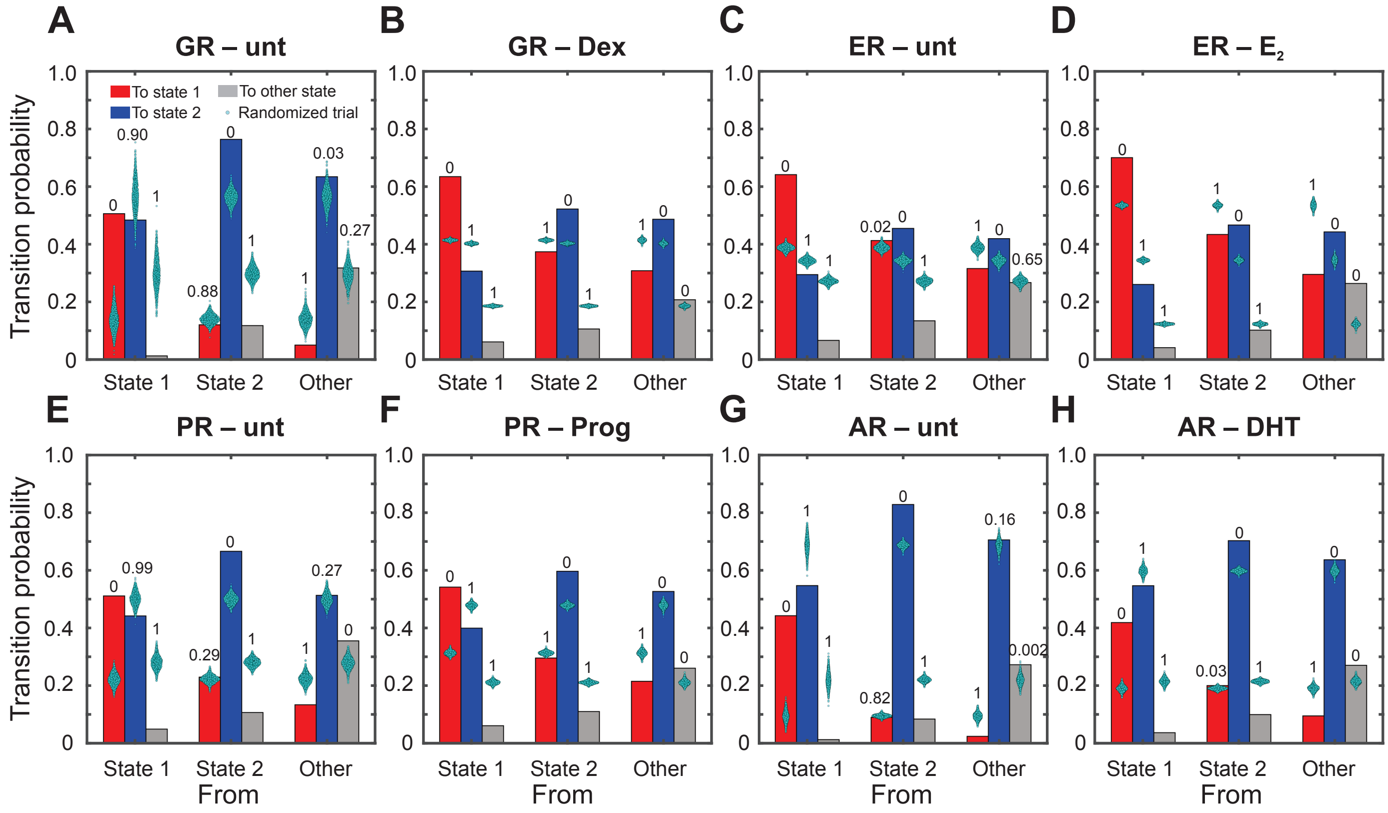
A
GR – unt
B
GR – Dex
C
ER – unt
D
ER – E_2
E
PR – unt
F
PR – Prog
G
AR – unt
H
AR – DHT
Transition probability
To state 1
To state 2
To other state
Randomized trial
State 1
State 2
Other
From

Figure S7

Fig. S7: Transition probabilities for steroid receptors compared against randomized trials. Transition probabilities between states 1, 2, and "other". For simplicity, all the other states detected by pEMv2 were combined into a single state. The groups along the x-axis represent the transition probability from the indicated state while the colors of the bars represent transitions into state 1 (red), state 2 (blue), and other states (gray). Cyan swarmcharts represent transition probabilities calculated for 1000 randomized ensembles. Numbers above the bars indicate the proportion of randomized trials with transition probability higher than the corresponding calculated probability. **(A)** Glucocorticoid receptor (GR) – unliganded. **(B)** GR activated with dexamethasone (Dex). **(C)** Estrogen receptor (ER) – unliganded. **(D)** ER activated with 17β-estradiol (E_2). **(E)** Progesterone receptor (PR) – unliganded. **(F)** PR activated with progesterone (Prog). **(G)** Androgen receptor (AR) – unliganded. **(H)** AR activated with dihydrotestosterone (DHT).

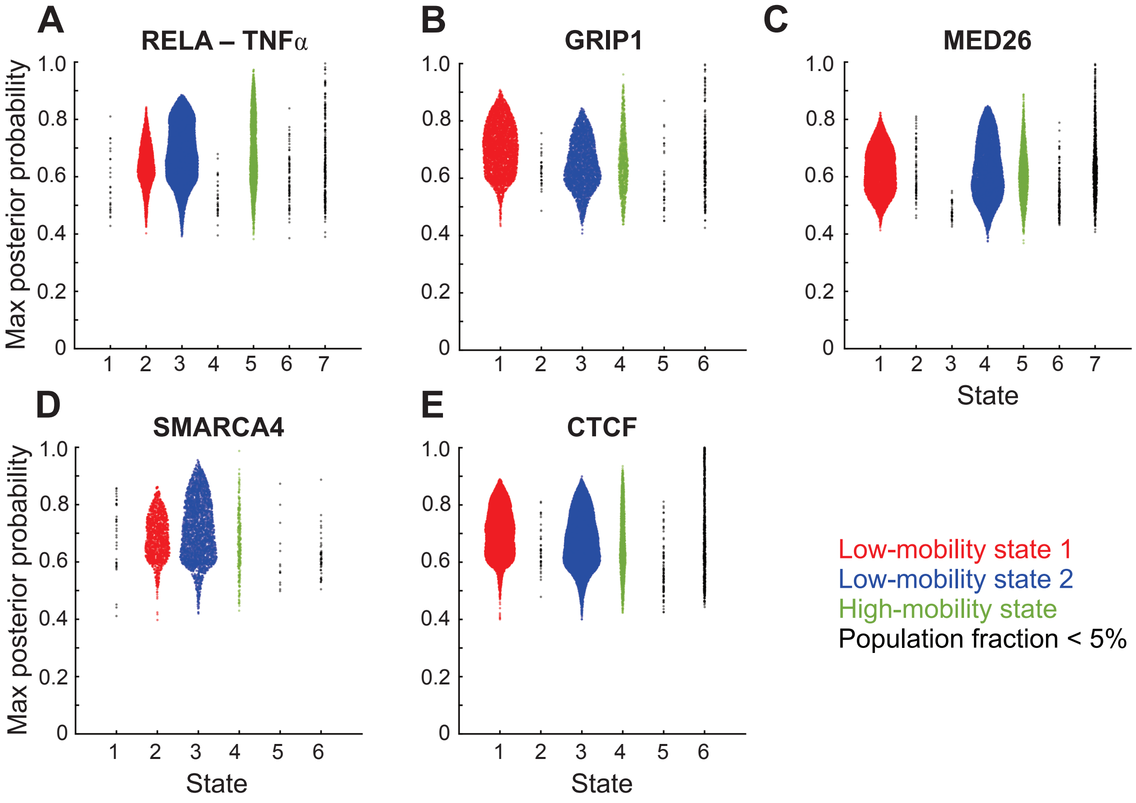
A
RELA – TNFα
B
GRIP1
C
MED26
D
SMARCA4
E
CTCF
Max posterior probability
State
1.0
0.8
0.6
0.4
0.2
0
1 2 3 4 5 6 7
Low-mobility state 1
Low-mobility state 2
High-mobility state
Population fraction < 5%

Figure S8

Fig. S8: Swarmcharts of maximum posterior probability as a function of states identified by pEMv2. Maximum posterior probability vs pEMv2 state for **(A)** RELA activated with TNFα, **(B)** GRIP1, **(C)** MED26, **(D)** SMARCA4, **(E)** CTCF.

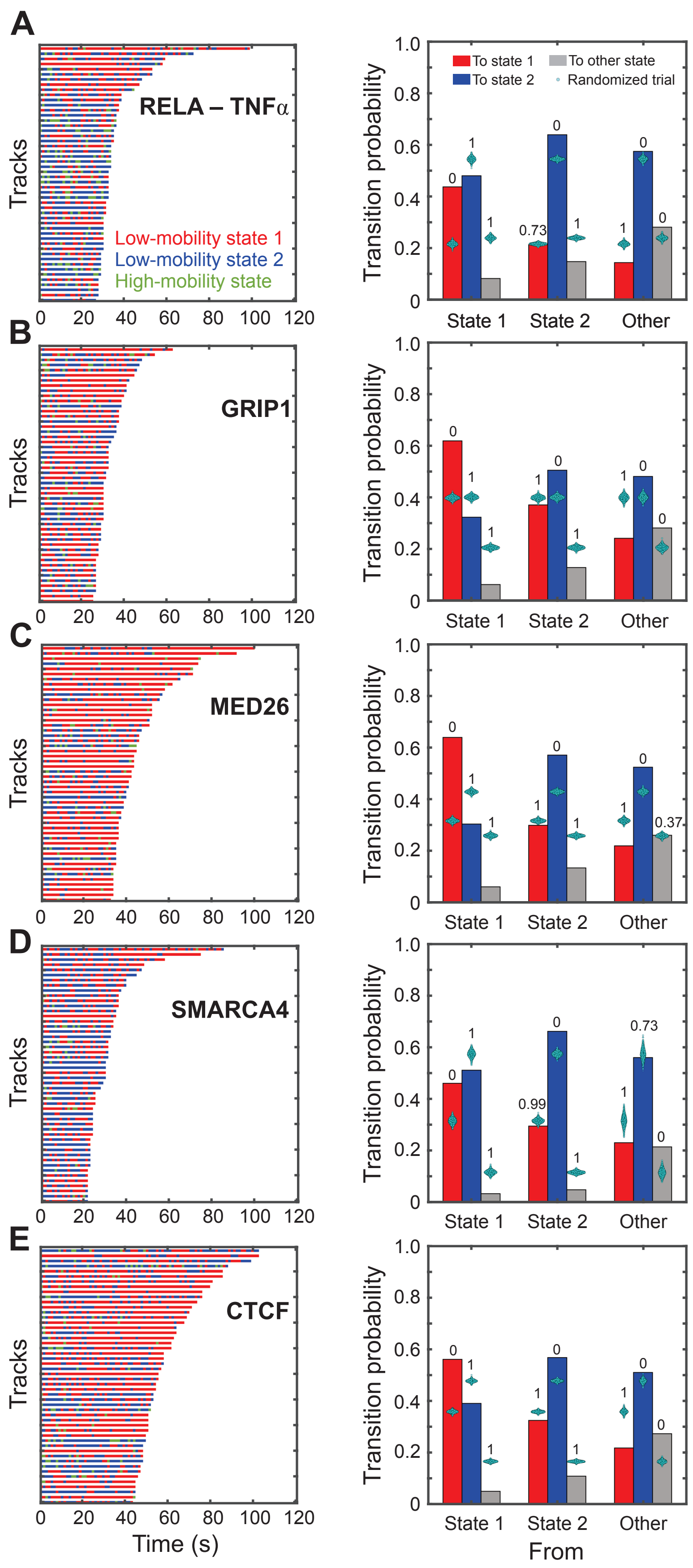
A
RELA – TNFα
Low-mobility state 1
Low-mobility state 2
High-mobility state
To state 1
To state 2
To other state
Randomized trial
B
GRIP1
C
MED26
D
SMARCA4
E
CTCF
Tracks
Time (s)
Transition probability
State 1
State 2
Other
From

Figure S9

Fig. S9: All tested coregulators exhibit switching between mobility states. For all panels: (left) Temporal reconstruction for 50 longest tracks color-coded by state assignment. (right) Transition probabilities between states 1, 2, and "other". For simplicity, all the other states detected by pEMv2 were combined into a single state. The groups along the x-axis represent the transition probability from the indicated state while the colors of the bars represent transitions into state 1 (red), state 2 (blue), and other states (gray). Cyan swarmcharts represent transition probabilities calculated for 1000 randomized ensembles. Numbers above the bars indicate the proportion of randomized trials with transition probability higher than the corresponding calculated probability. **(A)** RELA treated with TNFα, **(B)** GRIP1, **(C)** MED26, **(D)** SMARCA4, **(E)** CTCF.

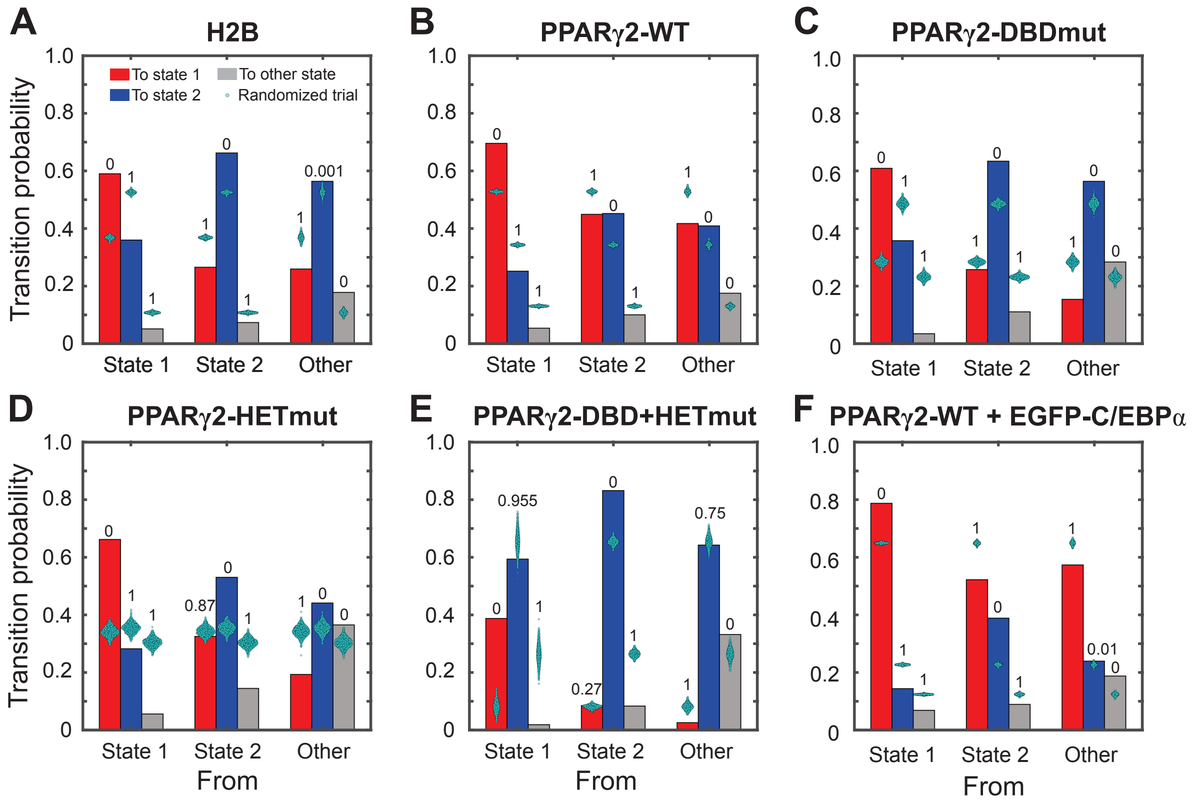

A
H2B
B
PPARγ2-WT
C
PPARγ2-DBDmut
D
PPARγ2-HETmut
E
PPARγ2-DBD+HETmut
F
PPARγ2-WT + EGFP-C/EBPα
To state 1
To state 2
To other state
Randomized trial
Transition probability
From
State 1
State 2
Other

Figure S10

Fig. S10: Transition probabilities for PPARγ2 mutants and H2B in 3T3-L1 pre-adipocytes. Transition probabilities between states 1, 2, and "other". For simplicity, all the other states detected by pEMv2 were combined into a single state. The groups along the x-axis represent the transition probability from the indicated state while the colors of the bars represent transitions into state 1 (red), state 2 (blue), and other states (gray). Cyan swarmcharts represent transition probabilities calculated for 1000 randomized ensembles. Numbers above the bars indicate the proportion of randomized trials with transition probability higher than the corresponding calculated probability. **(A)** H2B, **(B)** PPARγ2-WT, (C) PPARγ2-DBDmut, **(D)** PPARγ2-HETmut, **(E)** PPARγ2-DBD+HETmut, **(F)** PPARγ2-WT + EGFP-C/EBPα.

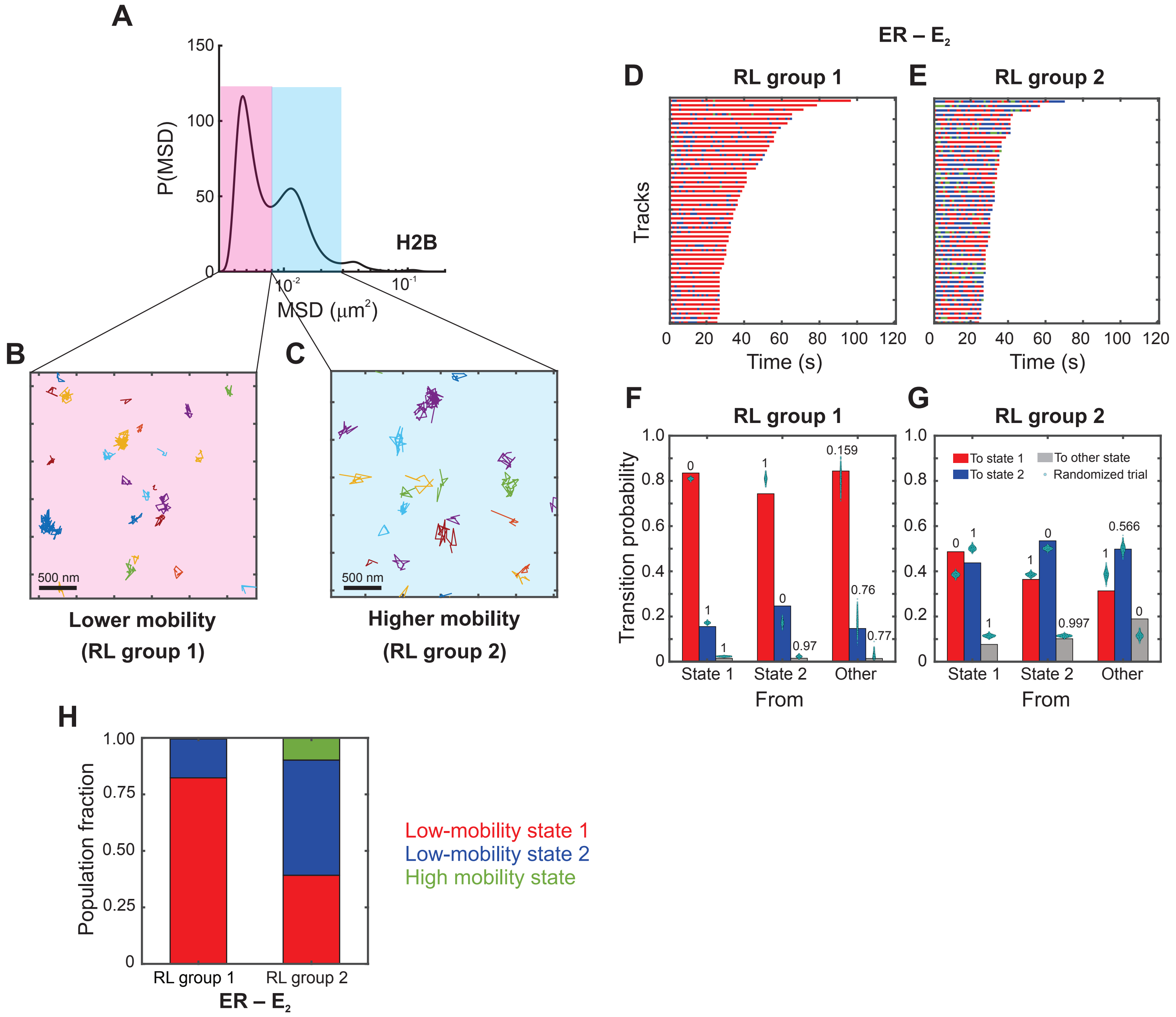
A
B
C
D
E
F
G
H
P(MSD)
MSD (μm²)
H2B
500 nm
Lower mobility
(RL group 1)
Higher mobility
(RL group 2)
ER – E₂
RL group 1
RL group 2
Tracks
Time (s)
Transition probability
To state 1
To state 2
To other state
Randomized trial
State 1
State 2
Other
From
Population fraction
RL group 1
RL group 2
ER – E₂
Low-mobility state 1
Low-mobility state 2
High mobility state

Figure S11

Fig. S11: Classifying tracks based on MSD distribution. (A) Minima in the predicted mean-squared distribution (MSD) distribution can be used to classify trajectories into two groups: tracks with an overall lower and higher mobility at 0.8 s. H2B tracks with an MSD in the magenta region are classified into the lower mobility group (RL group 1) while those with an MSD in the cyan region are classified into the higher mobility group (RL group 2). **(B)** Representative trajectories classified into RL group 1. **(C)** Representative trajectories classified into RL group 2. **(D – H)** Quantification of estrogen receptor (ER) activated by 17β-estradiol (E_2). **(D, E)** Temporal reconstruction of the 50 longest tracks of single molecules belonging to **(D)** RL group 1 (overall lower mobility) and to **(E)** RL group 2 (overall higher mobility). The tracks are color-coded to show the pEM identified states of the 1.2 s segments making up entire track. State 1 is depicted in red and state 2 in blue. Higher mobility states are colored green. **(F, G)** Transition probabilities calculated for molecules in **(F)** RL group 1 and **(G)** RL group 2. Transitions into pEM state 1 are shown in red, those into state 2 are shown in blue, and others in gray. Cyan swarmcharts show the results of the transition probability calculation for 1000 randomly permuted ensembles. Numbers above the bars display the proportion of these trials with a transition probability higher than the respective calculated transition probability. **(H)** Fraction of tracks in pEM state 1 (red), pEM state 2 (blue) and pEM state 3 (green) for trajectories classified into RL groups 1 and 2.

Cell line	Protein + treatment	% 2nd NN within 416 nm
**3617**	H2B	0.58%
**3617**	AR – unt	0.38%
**3617**	AR – DHT	1.54%
**3617**	CTCF	0.89%
**3617**	ER – unt	0.87%
**3617**	ER – E_2	0.76%
**3617**	GR – unt	0.24%
**3617**	GR – Dex	0.93%
**3617**	GRIP1	1.19%
**3617**	MED26	1.20%
**3617**	PR – unt	0.52%
**3617**	PR – Prog	1.01%
**3617**	RELA – TNFα	0.96%
**3617**	SMARCA4	0.29%
**3T3-L1**	H2B	0.60%
**3T3-L1**	PPARγ2-WT	0.74%
**3T3-L1**	PPARγ2-DBDmut	0.47%
**3T3-L1**	PPARγ2-HETmut	0.55%
**3T3-L1**	PPARγ2-DBD+HETmut	0.38%
**3T3-L1**	PPARγ2-WT + EGFP-C/EBPα	0.44%

Table S1

Table S1: Estimation of tracking errors. Tracking errors can occur if the labeling density is too high. The table indicates the percentage of tracked molecules that have two molecules within the maximum allowed 4 pixel jump distance in the subsequent frame. NN = nearest neighbor.

Cell line	Protein + treatment	% of tracks with ≥ 3 sub-tracks	% of sub-tracks belonging to tracks with ≥ 3 sub-tracks
**3617**	H2B	33%	76%
**3617**	AR – unt	27%	69%
**3617**	AR – DHT	24%	62%
**3617**	SMARCA4	23%	63%
**3617**	CTCF	31%	74%
**3617**	ER – E_2	29%	71%
**3617**	ER – unt	22%	61%
**3617**	GRIP1	29%	70%
**3617**	MED26	23%	63%
**3617**	PR – Prog	28%	71%
**3617**	PR – unt	20%	59%
**3617**	RELA – TNFα	26%	65%
**3617**	GR – unt	22%	61%
**3617**	GR – Dex	26%	66%
**3T3-L1**	H2B	36%	76%
**3T3-L1**	PPARγ2-DBD+HETmut	20%	61%
**3T3-L1**	PPARγ2-DBDmut	24%	66%
**3T3-L1**	PPARγ2-HETmut	20%	60%
**3T3-L1**	PPARγ2wt + EGFP-C/EBPα	31%	75%
**3T3-L1**	PPARγ2wt	28%	70%
	**Average**	**26 ± 5%**	**67 ± 6%**

Table S2

Table S2: Fraction of tracks that have at least three sub-tracks. Transition probabilities are calculated for those tracks that have at least three sub-tracks. This table lists the fraction of tracks that have at least three sub-tracks ("% of tracks with ≥ 3 sub-tracks"). In addition to this, "% of sub-tracks belonging to tracks with ≥ 3 sub-tracks" list the number of sub-tracks that belong to tracks that contain at least three sub-tracks, as a fraction of the total number of sub-tracks.

Movie S1. Representative single-molecule tracking movies.

Representative single-molecule tracking movies of two H2B-Halo expressing 3617 cells. Images are collected every 200 ms with 10 ms exposure. Scale bar 4 μm.

Movies S2 – S4. Molecules switch between two low-mobility states.

Representative H2B movie overlaid with tracks. The tracks are color-coded according to the mobility state they are assigned to by pEMv2. Red represents state 1 and blue represents state 2. Scale bar 500 nm.
